# Zn(II)–curcumin prevents cadmium-aggravated diabetic nephropathy by regulating gut microbiota and zinc homeostasis

**DOI:** 10.3389/fphar.2024.1411230

**Published:** 2024-06-05

**Authors:** Wenjia Sun, Xueting Mei, Jiasheng Wang, Zhicong Mai, Donghui Xu

**Affiliations:** ^1^ Laboratory of Traditional Chinese Medicine and Marine Drugs, Institute of Aquatic Economic Animals and Guangdong Provincial Key Laboratory for Aquatic Economic Animals, School of Life Sciences, Sun Yat-sen University, Guangzhou, China; ^2^ Laboratory Animal Center, Sun Yat-sen University, Guangzhou, China

**Keywords:** cadmium, diabetic nephropathy, Zn(II)–curcumin, gut microbiota, zinc homeostasis, metabolism

## Abstract

**Background:** Diabetic nephropathy (DN) is known as the most common complication of diabetes, resulting from a complex inheritance-environment interaction without effective clinical treatments. Herein, we revealed the protective effects and mechanisms of Zn(II)-curcumin, a curcumin derivative, against streptozotocin-induced DN in rats in the presence or absence of cadmium exposure.

**Methods:** The present study focused on investigating the therapy of Zn(II)-curcumin against cadmium-aggravated DN by regulating gut microbiota, metabolism, inflammation and zinc homeostasis based on pathological changes, TLR4/NF-κB signaling pathway, inductively coupled plasma-mass spectrometry (ICP-MS), 16S rRNA gene sequencing and gas chromatography-mass spectrometer (GC-MS).

**Results:** We found Zn(II)-curcumin significantly mitigated the cadmium-aggravated phenotypes of diabetic nephropathy, as indicated by the remission of renal dysfunction, pathological changes, inflammation and zinc dyshomeostasis in streptozotocin-treated rats exposed to cadmium. Administration of Zn(II)-curcumin significantly alleviated the dysbiosis of gut microbiota and the changes of serum metabolite profiles in rats treated with streptozotocin in combination with cadmium. Notably, fecal microbial transplantation identified the ability of Zn(II)-curcumin to regulate renal function, inflammation and zinc homeostasis was partly dependent on the gut microbiota.

**Conclusion:** These findings revealed that Zn(II)-curcumin alleviated cadmium-aggravated diabetic nephropathy by reshaping the gut microbiota and zinc homeostasis, which provided unique insights into the mechanisms of the treatment and prevention of diabetic nephropathy.

## 1 Introduction

Diabetic nephropathy is a major complication of diabetes with the incidence of approximately 30%–47% in all diabetic patients and represents a major cause of the increased risks of developing renal failure, cardiovascular events, and death (Jiang et al., 2020; Defronzo et al., 2021; Xing et al., 2021). Structural changes in diabetic nephropathy in type 1 and type 2 diabetes are mainly in the glomerulus with mesangial expansion and thickening of the glomerular basement membrane (GBM), and they also include arteriolar, tubular, and interstitial lesions (Navarro-Gonzalez et al., 2011). Diabetic nephropathy is characterized by hyperglycemia, hyperlipidemia, and progressive deterioration of renal function, which are the main phenotype bases for the clinical treatment (Song et al., 2022). Currently, clinical strategies to alleviate the progression of diabetic nephropathy are limited. The treatment options such as angiotensin II receptor blockers (ARB), sodium–glucose cotransporter-2 (SGLT-2) inhibitors, and angiotensin-converting enzyme (ACE) inhibitors have received much attention for their anti-inflammatory and cardio-protective activities, but their risks and benefits need to be clarified (Fineberg et al., 2013; Hu et al., 2020; Huang et al., 2022; He et al., 2023; Shen et al., 2023). The development and progression of diabetic nephropathy involve multiple factors, including inheritance and environment, resulting in the underlying pathogenesis that is still not fully understood. Thus, it is very necessary to further discover the mechanisms and develop new drugs for the treatment of diabetic nephropathy.

Gut dysbiosis has been identified as an important cause of the development of diabetes and its complications (Fan and Pedersen, 2021). The disturbance in intestinal microbiota increases the leakage of pro-inflammatory bacterial products into the circulation (Cao et al., 2021a; Cao et al., 2021b). These harmful substances can cause insulin resistance and accelerate the progression of diabetes and its complications in patients. For instance, Gram-negative bacteria-produced endotoxin lipopolysaccharides (LPS) and peptidoglycans can stimulate the toll-like receptor 4 (TLR4)- and NF-κB-involved inflammatory response, leading to systemic and renal inflammation, which further worsen kidney dysfunction (Sun et al., 2020a; Yang et al., 2020; Zhou et al., 2023). The dsybiosis of the gut microbiota can be sensitively caused by western diet and environmental factors (Amato et al., 2015). For example, cadmium (Cd) exposure has been found to induce a remarkable alteration in the microbial community structure and composition, along with the activation of intestinal inflammatory response and intestinal barrier disruption (Tinkov et al., 2018). Furthermore, an environmental dose of Cd at early stages of life induces the alterations in gut microbiota, which accelerates hepatic lipid accumulation and results in lifelong metabolic syndrome, such as fat accumulation and hyperglycemia (Ba et al., 2017). Notably, Cd exposure has been positively linked to increased diabetes risk in several previous studies (Xing et al., 2018; He et al., 2024). However, the potential pathogenic mechanism of Cd exposure affecting diabetic nephropathy is still unclear, which prevents the development of new drugs for the prevention and treatment of diabetic nephropathy.

Plant-derived natural products and their derivatives represent effective potential treatment for type 2 diabetes and related metabolic diseases in the past decades (Qi et al., 2022; Wu et al., 2023; Hu et al., 2024). Curcumin, a natural small-molecule compound, is extracted from *Curcuma longa* Linn. (turmeric) and used as an anti-diabetic, anti-inflammatory, anticancer, and anti-aging agent in preclinical and clinical trials (Kotha and Luthria, 2019; Ghosh et al., 2015). However, the therapeutic usage of curcumin is limited due to the poor aqueous solubility and bioavailability (Prasad and Lall, 2022). To address these disadvantages, several curcumin formulations, including curcumin–metal complexes and solid dispersion, have been developed in our previous studies. Zn(II)–curcumin (ZnCM) not only exerts stronger anti-cancer, anti-cardiotoxic, and anti-diabetic effects than curcumin alone but also significantly attenuates gut dysbiosis and zinc dyshomeostasis in animals (Wu et al., 2019a; Wu et al., 2019b). Therefore, we hypothesize that oral consumption of ZnCM could alleviate the dysregulation of the gut microbiota and prevent the development of diabetic nephropathy.

In the present study, we first assessed the impact of Cd exposure on streptozotocin (STZ)-induced diabetic nephropathy by determining the renal function, inflammation, and zinc homeostasis in rats. Next, the efficacy of ZnCM on Cd-aggravated diabetic nephropathy was evaluated in STZ-treated rats. 16S rRNA gene sequences and gas chromatography–mass spectrometry (GC-MS) were performed to analyze the gut microbiota composition and serum metabolite profiling, respectively. The role of gut microbiota in the mitigation of ZnCM on diabetic nephropathy was confirmed by fecal microbiota transplantation.

## 2 Methods

### 2.1 Materials and chemicals

Zn(II)–curcumin (ZnCM) was synthesized from curcumin (Guangdong Zhongda Greenfield Biotech. Co., Guangzhou, China) and zinc acetate, and prepared as solid dispersion using PVP-30 (BASF Chemical Ltd., Atlantic City, NJ, United States), according to our previous descriptions (the mass ratio for ZnCM: PVP-30 = 1 : 6) (Mei et al., 2011). Streptozotocin (STZ; S0130) was purchased from Sigma-Aldrich Corporation (Sigma-Aldrich, St. Louis, MaO, United States). Other reagents were of analytical grade and were purchased from local chemical suppliers.

### 2.2 Animal experimental design

**FIGURE 1:**
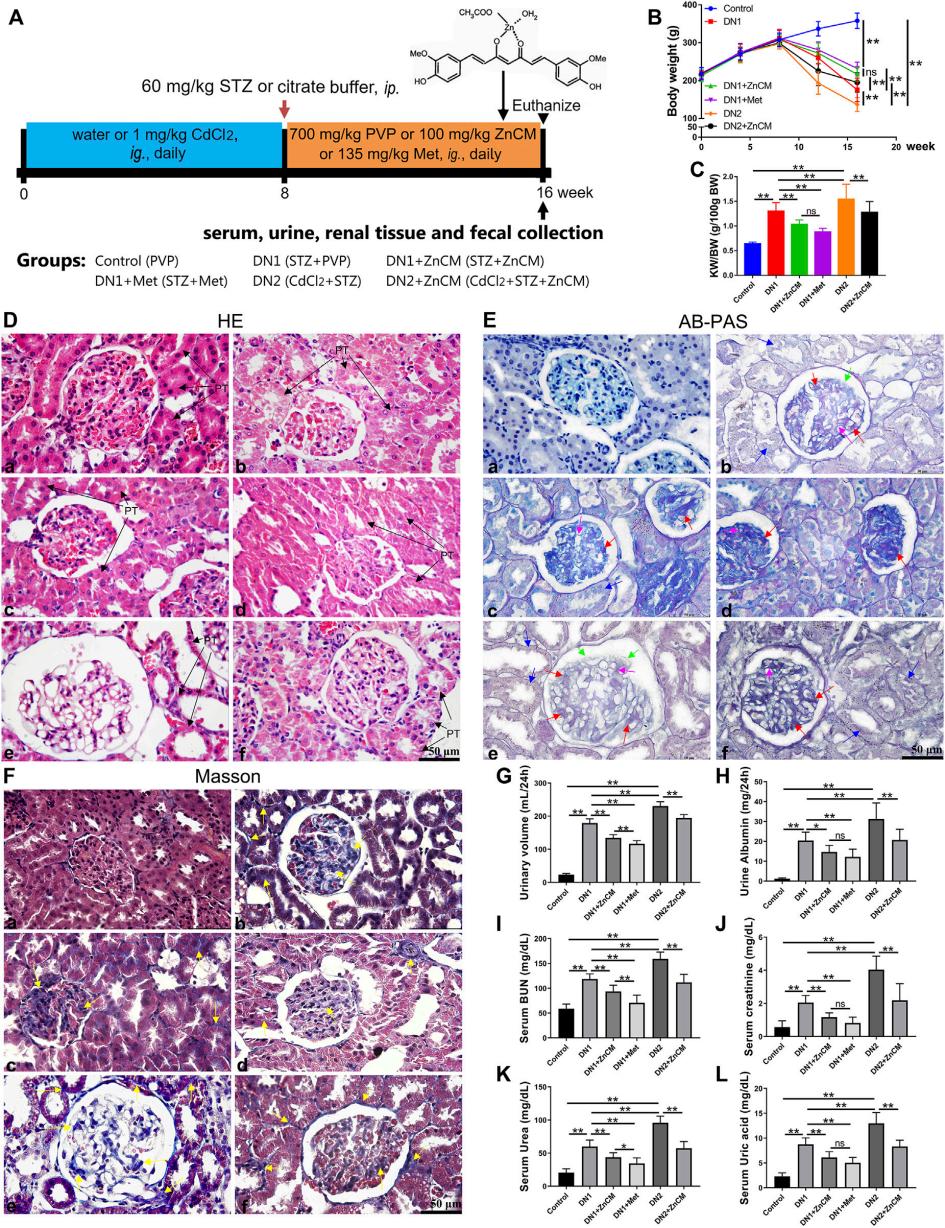
ZnCM attenuated renal pathological deterioration and dysfunction in STZ-induced rats exposed to Cd. **(A)** Schematic diagram of the experimental design. **(B)** Body weight throughout the experiment. **(C)** The ratio of kidney weight to body weight (KW/BW). **(D–F)** Representative images of H&E staining, AB-PAS staining, and Masson’s trichrome staining of the kidney (scale bar, 50 μm). PT: proximal tubule; proximal tubule epithelial cell edema and hypertrophy (black arrowheads); tubular epithelial damage (blue arrowheads); thickening of the glomerular basement membrane (light purple arrowheads); mesangial regions proliferative and expansion (red arrowheads); podocyte apoptosis and necrosis (green arrowheads); fibroblasts proliferation (yellow arrowheads). **(G, H)** Levels of urinary volume and albumin of 24 h. **(I)** Levels of blood urea nitrogen (BUN). **(J–L)** Levels of creatinine, urea, and uric acid in the serum. Data are represented by mean ± SD (*n* = 8) and were analyzed by one-way ANOVA followed by the original FDR method of the Benjamini and Hochberg test. *p < 0.05 and **p < 0.01; ns indicates no significance.

All animal experiments were approved by the Experimental Animal Care and Use Committee of Sun Yat-sen University (number IACUC-20221030002) and strictly conducted in line with the Guidelines for Care and Use of Laboratory Animals of this organization. Healthy adult female Sprague–Dawley rats (body weight 240–260 g) were provided by the Laboratory Animal Center of Sun Yat-sen University. They were housed under specific pathogen-free standard conditions with a 12-h light/dark cycle and free access to diet and water. After acclimation for 1 week, animals were treated in the presence or absence of 1 mg/kg CdCl_2_ in drinking water in the initial 8 weeks, subsequently, treated with or without a single intraperitoneal injection of STZ (60 mg/kg) (Xu et al., 2020), and orally administered with or without 100 mg/kg/d ZnCM, 700 mg/kg/d PVP, or 135 mg/kg/d Met (metformin, positive drug) for 8 weeks (*n* = 8 per group). The schematic diagram of the experimental design is shown in Figure 1A. The animals were weighed weekly in the course of the experiments. In addition, the stool samples were collected at week 16 for gene analysis. At the end of the experiment, rats were fasted for approximately 12 h and humanely euthanized by the administration of CO_2_. Afterward, serum, urine, and kidney samples were collected for biochemical, histopathological, and metabolic analyses, respectively.

### 2.3 Fecal microbiota transplantation (FMT)

Recipient rats were pretreated with a cocktail of antibiotics (ABX containing 1 g/L ampicillin, 0.5 g/L neomycin, 0.5 g/L vancomycin, and 1 g/L metronidazole) in drinking water for 1 week. Fresh feces from donor rats were daily collected, pooled (100 mg feces/mL), and vigorously mixed for 30 s, and then centrifuged at 800 g for 3 min. A measure of 100 μL of fecal supernatants of each rat was orally transferred to STZ-treated recipient rats once a day for 8 weeks. Kidney, blood, and urine samples from the recipient rats were collected for subsequent experiments.

### 2.4 Biochemical analysis

The levels of creatinine, uric acid, urea, and urea nitrogen (BUN) in the serum or urine were tested using commercial kits (Nanjing Jiancheng Bioengineering Institute, Nanjing, China) (Xu et al., 2020). The contents of serum TNF-α, IL-1β, and IL-6 were quantified using ELISA kits (Nanjing Jiancheng Bioengineering Institute, Nanjing, China), according to the instructions of reagents and previous methods (Zhang et al., 2020; Li W. et al., 2021; Zhang et al., 2021).

### 2.5 Histopathological analysis

Renal tissue was analyzed histologically as previous methods with minor modifications (Wang et al., 2021; Hao et al., 2022). Briefly, renal tissues were fixed in 4% paraformaldehyde and embedded in paraffin. A measure of 3-μm sections were stained with hematoxylin and eosin (H&E), Alcian blue-periodic acid–schiff (AB-PAS), or Masson’s trichrome staining by using standard methods (Zhou et al., 2021; Tao et al., 2022; Zhang et al., 2023). Images were captured under a Leica DM6B microscope (Leica, Weltzar, Germany).

### 2.6 Western blotting

The expressions of proteins were determined by Western blot as described before (Ding et al., 2021; Chen et al., 2022; Zhou et al., 2022). In brief, the total protein of renal tissue was extracted by using RIPA extraction buffer, and the concentrations were quantified using BCA protein assay kits. 10% SDS polyacrylamide gel was used to separate proteins, transferred onto PVDF membranes, and then blocked with 5% skimmed milk for 1 h at room temperature. The primary antibodies (showed in Supplementary Table S1) were immunoblotted with the membranes at 4°C overnight and followed by the incubation with the respective secondary antibodies for 1 h at room temperature. The bands were visualized using ECL kits (Applygen, Beijing, China) on the Tanon5200 chemiluminescence imaging system (Shanghai Tianneng Technology Co., Ltd., Shanghai, China). The relative expressions of proteins were calculated against β-actin.

### 2.7 Zn and Cd measurements

For the quantification of Zn and Cd, 0.1–0.5 g of renal tissue was digested by concentrated nitric acid in a microwave digestion system (MARS-6, CEM Co. Ltd., Matthews, NC, United States). The obtention was subjected to determine the contents of Zn and Cd by using inductively coupled plasma-mass spectrometry (ICP-MS; Thermo Fisher Scientific, Waltham, MA, United States) (Wu et al., 2019a; Wu et al., 2019b). The following formula can be used to calculate the Zn or Cd content per gram of tissue:

Zn or Cd content (μg/g) = (Zn or Cd concentration of tissue suspension) × liquid volume (mL)/wet weight of tissue.

For the imaging of Zn ions in renal tissue, the sections were deparaffinized, rehydrated, and incubated with fluorescent Zn ion probe TSQ (AAT Bioquest, Inc., United States) for 30 min at room temperature (Wu et al., 2019a; Wu et al., 2019b). Images were captured using a confocal laser scanning microscope (LSM880, Zeiss, Germany).

### 2.8 16S rRNA gene amplicon sequencing analysis

The composition of fecal microbiota was analyzed by 16S rRNA-sequencing, as previously described (Li Y. et al., 2021; Wang et al., 2023). In brief, genomic DNA of bacteria was extracted from feces using the QIAamp DNA Stool Mini Kit (QIAGEN, Hilden, Germany) according to the manufacturer’s instructions. DNA concentration and purification were determined by using a NanoDrop instrument (Thermo Fisher Scientific, Waltham, MA, United States). The V3–V4 regions of genes were amplified with the following primers: 338F (5′-ACT​CCT​ACG​GGA​GGC​AGC​AG-3′) and 806R (5′-GGACTACHVGGGTWTCTAAT-3′) using the thermocycler PCR system (thermal cycling condition: 95°C for 5 min (1 cycle), 95°C for 30 s, 50°C for 30 s, 72°C for 40 s (25 cycles), and a final extension at 72°C for 7 min). After purification and quantification, the amplicons were subjected to sequence on a HiSeq 2500 platform (Illumina, San Diego, United States) with the standard protocols by Biomarker Technologies Co., Ltd. (Beijing, China) (Li et al., 2023). The alpha diversity, beta diversity, and differential types of bacteria were analyzed with an online platform at BMKCloud (www.biocloud.net).

### 2.9 Metabolite analysis

The serum metabolites were extracted and measured by gas chromatography–mass spectrometry (GC-MS), as previously described with minor modifications (Chen et al., 2019; Jiang et al., 2021). A measure of 80 μL of 20 mg/mL methoxyamine hydrochloride (Sigma-Aldrich, St Louis, MO, United States) in pyridine was added in 100 μL of serum. The mixture was vigorously vortexed for 5 min and incubated at 37°C for 90 min. A measure of 80 μL of N-methyl-N-trimethylsilyltrifluoroacetamide (Sigma-Aldrich, St Louis, MO, United States) was added and derivatized at 37°C for 30 min. The mixture was centrifuged at 30,000 × g at 4°C for 15 min, and the supernatant was filtered through a 0.2-μM filter. The filtrates were lyophilized and then resuspended in ultrapure water. The metabolites were detected by using an Agilent GC7890A-MS5975C gas chromatography–mass spectrometer (Agilent Technologies, Santa Clara, CA, United States) equipped with the experienced instrument settings (described in Supplementary Table S2) and coupled to an LCMS-8050 triple-quadrupole mass spectrometer (Shimadzu Corporation, Kyoto, Japan). The data were analyzed using SIMCA 14.1 software (Umetrics, Umea, Sweden). Spearman’s correlation between differential metabolites and microbiota was analyzed by using GraphPad Prism software 9.0 or MetaboAnalyst 5.0 (https://www.metaboanalyst.ca/).

### 2.10 Statistics

GraphPad Prism software 9.0.0 (GraphPad Software, La Jolla, CA, United States) was used for the statistical analyses. Unpaired two-tailed t-tests were used to determine the significance of the differences between two groups. One-way ANOVA or two-way ANOVA was used to compare the statistical significance among more than two groups followed by the original false discovery rate (FDR) method of Benjamini and Hochberg or Tukey’s multiple comparison tests. Data are expressed as means ± standard deviation (SD). *p*-values <0.05 were statistically significant.

## 3 Results

### 3.1 ZnCM improves the renal function in STZ-induced rats exposed to Cd

To test whether ZnCM could protect the renal function of diabetic rats, STZ-induced rats were orally gavaged with 100 mg/kg ZnCM for 8 weeks in the presence or absence of Cd exposure (Figure 1A). Compared with control rats, STZ treatment induced a significant decrease in the body weight of rats. Cd exposure decreased body weights of STZ rats, and this could be significantly prevented by the administration of ZnCM (Figure 1B). Furthermore, compared with STZ-treated rats, the ratios of kidney weight/body weight (KW/BW) were significantly increased in rats exposed to Cd, which could be significantly reduced after ZnCM treatment (Figure 1C). Following this discovery, pathobiology and renal function parameters were analyzed. Compared with control rats, STZ treatment induced significant pathological changes in glomeruli, as evidenced by proximal tubule edema and hypertrophy with tubular epithelial cell damage, thickening of the glomerular basement membrane, mesangial region proliferation and expansion, podocyte apoptosis and necrosis, and the proliferation of the glomerular and glomeruli interstitial fibroblasts in rats (Figures 1D–F). Obviously, these changes in the kidney induced by STZ rats exposed to Cd were more serious than those by STZ alone. Notably, the administration of ZnCM dramatically ameliorated nephrotic phenotype in STZ-treated rats exposed to Cd, indicating that ZnCM effectively delayed the progression of diabetic nephropathy. Moreover, oral consumption of ZnCM not only significantly reduced the urinary volume and urine albumin but also remarkably decreased the levels of serum BUN, creatinine, urea, and uric acid in Cd-exposed rats with diabetic nephropathy induced by STZ (Figures 1G–L). These results indicate that ZnCM could effectively alleviate renal pathological deterioration and dysfunction in Cd-exposed rats with diabetic nephropathy.

### 3.2 ZnCM attenuates inflammatory response in Cd-exposed rats induced by STZ

**FIGURE 2:**
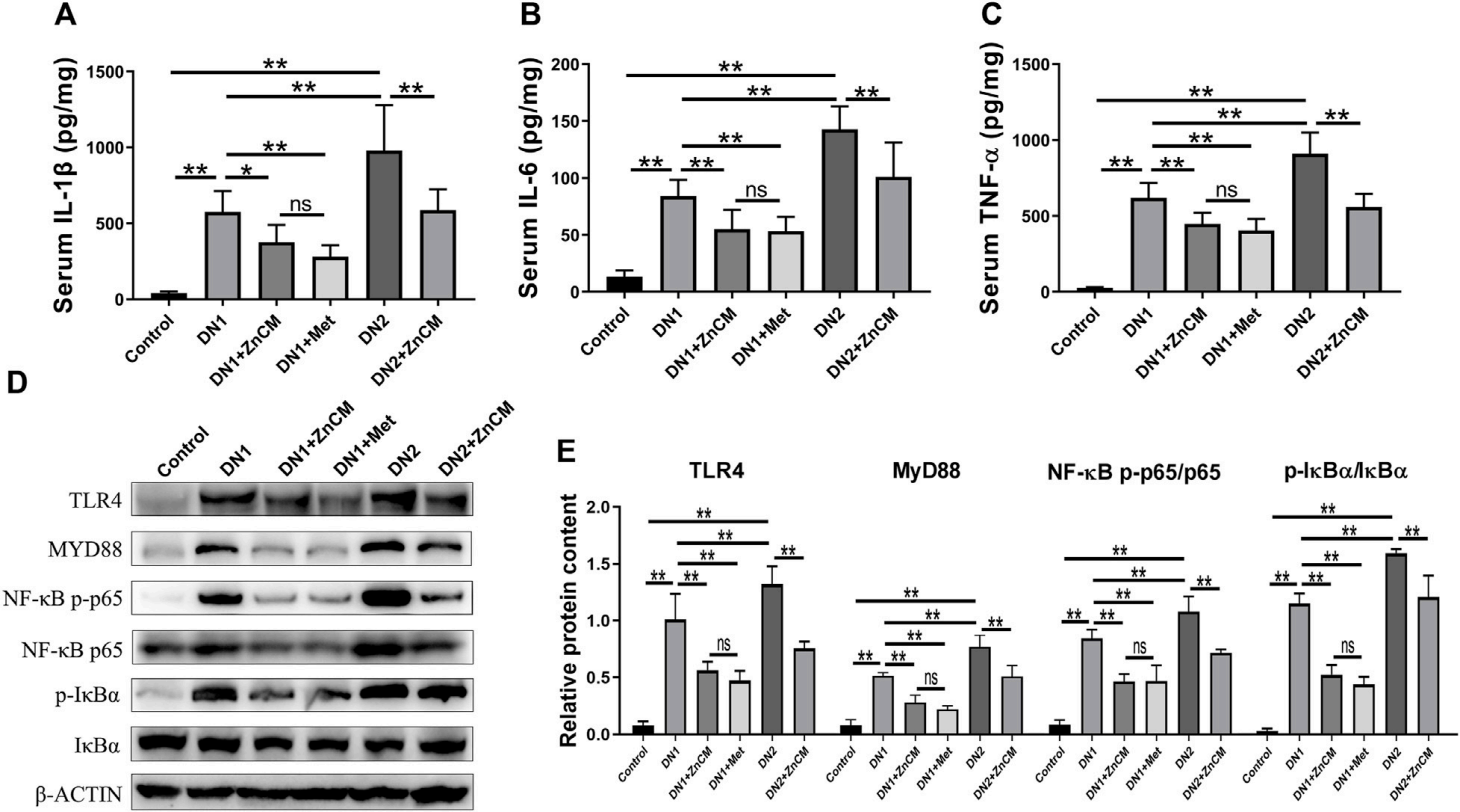
ZnCM relieved the inflammation in Cd-exposed rats with diabetic nephropathy. **(A–C)** The concentrations of IL-1β, IL-6, and TNF-α in the serum determined by ELISA (*n* = 8). **(D)** Representative blots of renal TLR4, MYD88, NF-κB p-p65, NF-κB p65, p-IκBα, and IκBα. **(E)** Relative expressions of TLR4, MYD88, NF-κB p-p65, NF-κB p65, p-IκBα, and IκBα quantified from (d) (*n* = 3). Data are represented by mean ± SD and analyzed by one-way ANOVA followed by the original FDR method of the Benjamini and Hochberg test. *p < 0.05 and **p < 0.01; ns indicates no significance.

As the pathology of diabetic nephropathy is associated with the overproduction of early response pro-inflammatory cytokines (He et al., 2022), we determined the inflammatory biomarkers (Sun et al., 2020b; Wang et al., 2020b). STZ treatment induced a significant increase in the levels of IL-1β, IL-6, and TNF-α (Figures 2A–C) in rats exposed to Cd when compared to those of control rats. ZnCM treatment significantly attenuated inflammatory response by decreasing those levels of response pro-inflammatory cytokines in STZ-treated rats exposed to Cd. STZ-treated rats exposed to Cd display significant increase in the protein expressions of TLR4, MYD88, NF-κB p-p65/NF-κBp65, and p-IκBα/IκBα in the kidney when compared with control rats. Interestingly, the administration of ZnCM clearly downregulated those increased protein expressions in STZ-treated rats exposed to Cd (Figures 2D, E). These findings provide evidence that treatment with ZnCM could attenuate inflammatory response and may be related to the inactivation of the TLR4/NF-κB pathway in Cd-exposed rats with diabetic nephropathy.

### 3.3 ZnCM attenuated Zn dyshomeostasis in Cd-exposed rats with diabetic nephropathy

**FIGURE 3:**
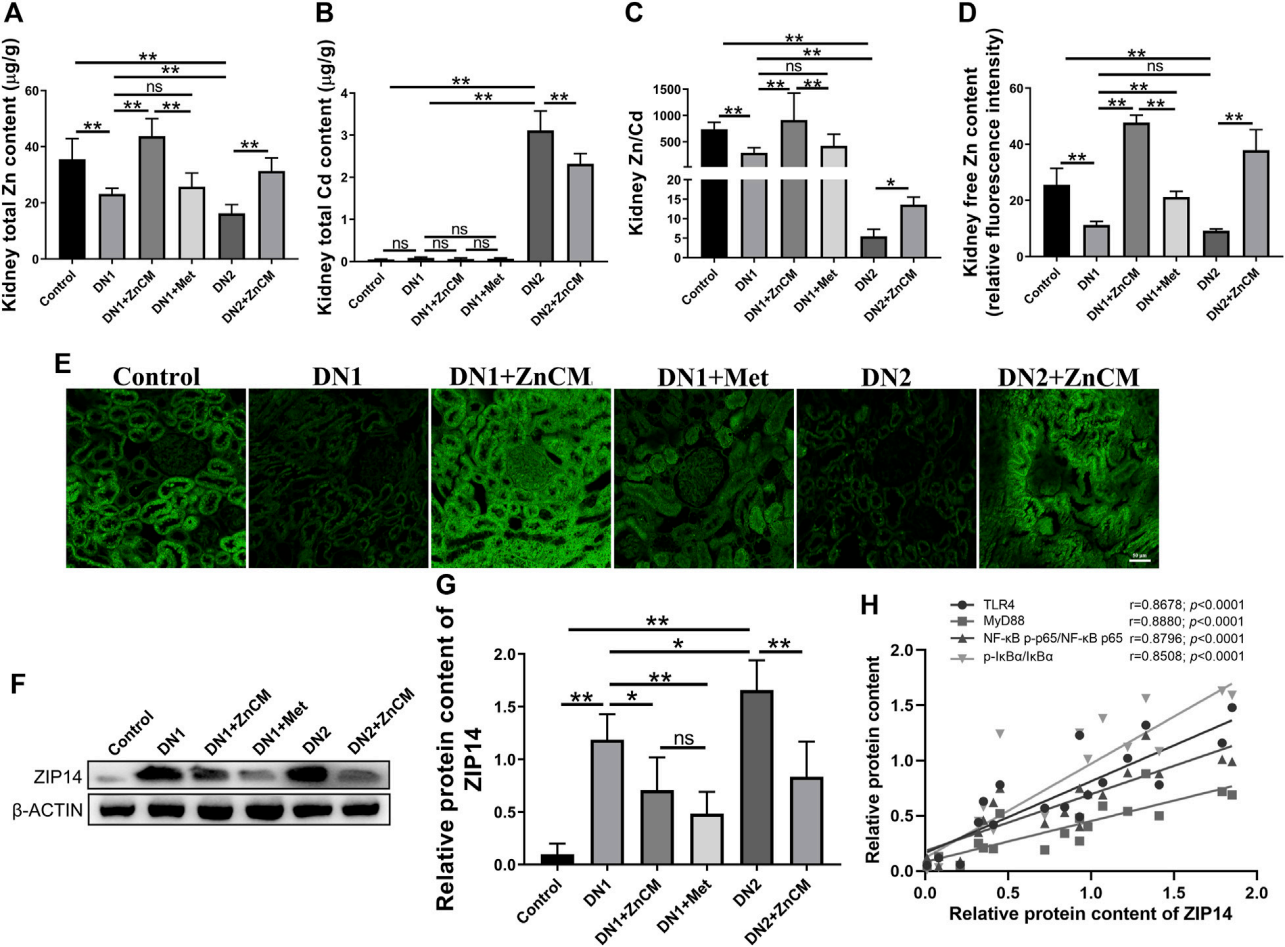
ZnCM relieved zinc dyshomeostasis in Cd-induced rats treated by STZ. **(A)** Renal Zn and **(B)** Cd levels were detected by ICP-MS (*n* = 8). **(C)** Zn/Cd ratio in the kidney (*n* = 8). **(D, E)** The level of free Zn quantified from fluorescence intensity. (*n* = 4). **(E)** Representative images of renal-free zinc level were determined by TSQ (scale bar, 50 μm). **(F, G)** Representative blots of renal ZIP14 and relative expressions (*n* = 3). **(H)** Relationships between the relative protein expression of ZIP14 and the relative expressions of TLR4, MYD88, NF-κBp-p65/NF-κBp65, and p-IκBα/IκBα. Data are represented by mean ± SD and analyzed by one-way ANOVA followed by the original FDR method of the Benjamini and Hochberg test or Spearman’s rank correlation. *p < 0.05 and **p < 0.01; ns indicates no significance.

Zinc homeostasis is often impaired in diabetic nephropathy (Al-Timimi et al., 2014). We were interested to test the changes of zinc in the kidney. A significant decrease in the level of total Zn and Zn/Cd ratio and a remarkable elevation of total Cd levels were observed in the kidneys of STZ-treated rats when compared with controls. Compared with STZ-treated rats, those changes in zinc homeostasis were significantly aggravated in those rats exposed to Cd, which could be effectively reversed after ZnCM treatment (Figures 3A–C). Compared to control rats, STZ-treated rats with or without Cd all had a lower level of renal-free Zn, which was significantly increased after ZnCM treatment (Figures 3D, E). Moreover, a remarkable increase in zinc transporter ZIP14 expression in STZ-treated rats exposed to Cd was observed when compared with either control rats or rats treated with STZ alone. Notably, treatment with ZnCM significantly reduced the expression of ZIP14 in Cd-exposed rats with diabetic nephropathy (Figures 3F, G). Furthermore, a significant positive correlation was presented between ZIP14 content and relative expressions of inflammation-related proteins, namely, TLR4, MYD88, NF-κBp-p65/NF-κBp65, and p-IκBα/IκBα (Figure 3H). Overall, these results suggest that Cd exposure significantly deteriorated Zn dyshomeostasis in diabetic rats, which could be effectively reversed by ZnCM supplementation.

### 3.4 ZnCM modulates gut microbiota dysbiosis in Cd-exposed diabetic nephropathy rats

**FIGURE 4:**
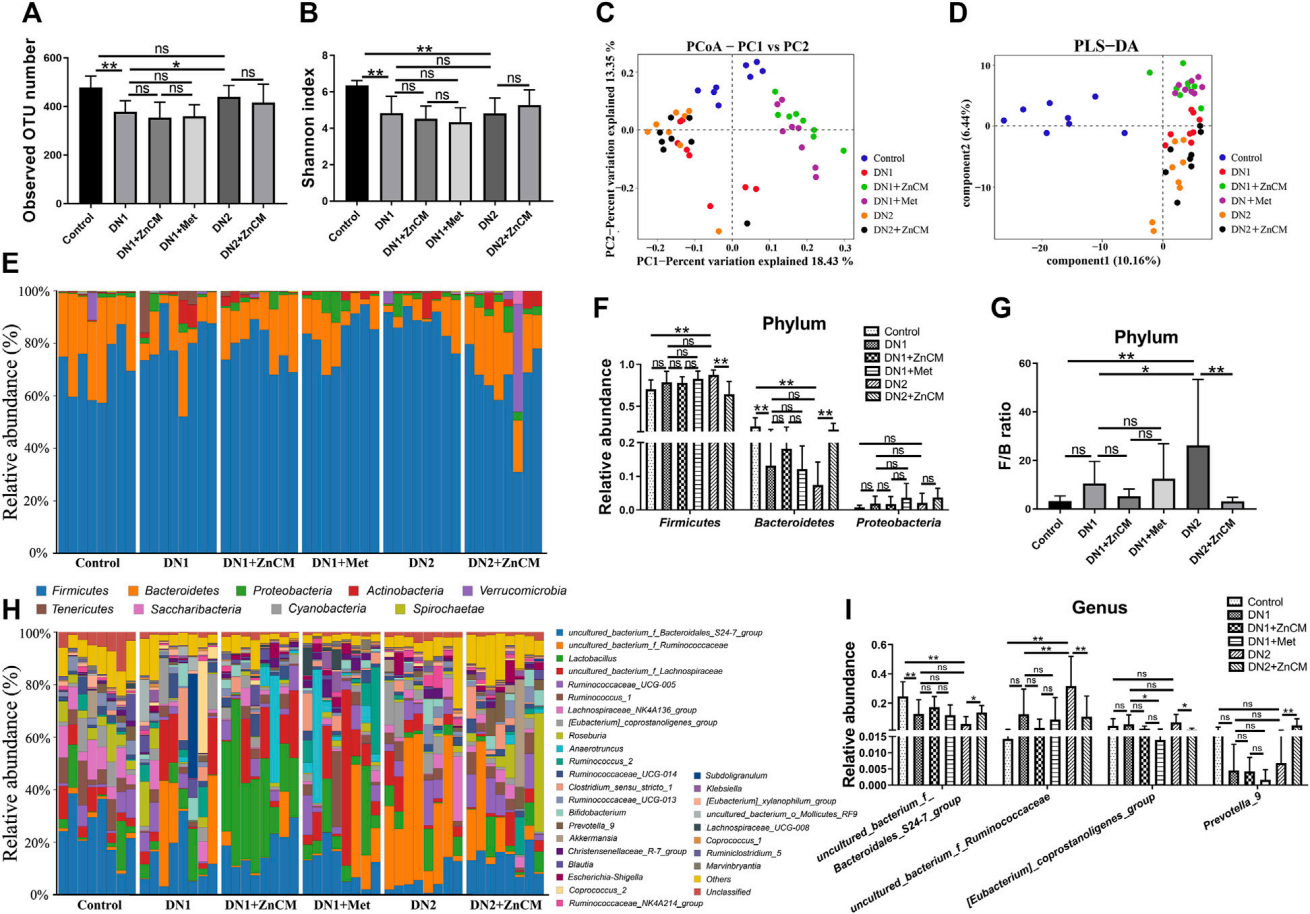
Oral administration of ZnCM regulated the composition of gut microbiota in Cd-induced diabetic nephropathy rats. **(A)** The number of observed OTU. **(B)** α-diversity of gut microbiota determined by the Shannon index. **(C)** PCoA and **(D)** PLS-DA of gut microbiota composition by weighted UniFrac distance (ANOSIM test). **(E)** The composition of the gut bacteria at the phylum level. **(F)** The relative abundance of *Firmicutes*, *Bacteroides*, and *Proteobacteria*. **(G)** The ratio of *Firmicutes*/*Bacteroides*. **(H)** The relative abundance of top 30 abundant bacteria at the genus level. **(I)** The relative abundance of major altered bacteria at the genus level. Data are shown as means ± SD (*n* = 8) and analyzed by one-way ANOVA followed by the original FDR method of the Benjamini and Hochberg test. *p < 0.05 and **p < 0.01; ns indicates no significance.

To further explore the effects of ZnCM on the gut microbiota in Cd-exposed rats with diabetic nephropathy, we performed microbial 16S rRNA gene sequencing for feces. Compared to controls, the observed OTU number and α-diversity (Shannon index) (Figures 4A, B) were significantly decreased in the rats with diabetic nephropathy independent of Cd exposure; β-diversity determined by principal coordinate analyses (PCoA) and partial least-squares discriminant analysis (PLS-DA) (Figures 4C, D) revealed that the gut microbial compositions of diabetic nephropathy rats were significantly different from those of control rats. In addition, ZnCM treatment significantly altered the composition of the gut microbiota in STZ-treated rats exposed to Cd or not (Figures 4C, D). At the phylum level, *Firmicutes*, *Bacteroidetes*, and *Proteobacteria* are the dominated phylum in the microbial community structure (Figures 4E–G). Compared with controls, STZ-treated rats exposed to Cd induced a significant increase in the relative abundance of *Firmicutes* and decrease in the relative abundance of *Bacteroidetes*, which were dramatically reversed by ZnCM supplementation. In addition, we found that STZ-induced rats had a highly increased *Firmicutes*-to-*Bacteroidetes* (F/B) ratio, a biomarker of gut dysbiosis, which was markedly reduced by ZnCM treatment (Figures 4F, G). At the genus level, compared with the control group, the relative abundance of *uncultured_bacterium_f_Bacteroidales_S24-7_group* (*S24-7_group*) and *Prevotella_9* were significantly decreased, and the relative abundance of *uncultured_bacterium_f_Ruminococcaceae*, and *[Eubacterium]_coprostanoligenes_group* were increased in STZ-treated rats exposed to Cd. These altered genera in Cd-exposed rats with diabetic nephropathy were markedly reversed by ZnCM supplementation. However, there is no significant difference in these microbiota between Met and ZnCM in both phylum and genus levels in rats with diabetic nephropathy (Figures 4H, I). These results show that Cd exposure destructed the composition of the gut microbiota in rats with diabetic nephropathy, which could considerably be reversed by ZnCM supplementation.

### 3.5 ZnCM altered metabolite profiles in Cd-induced diabetic nephropathy rats

**FIGURE 5:**
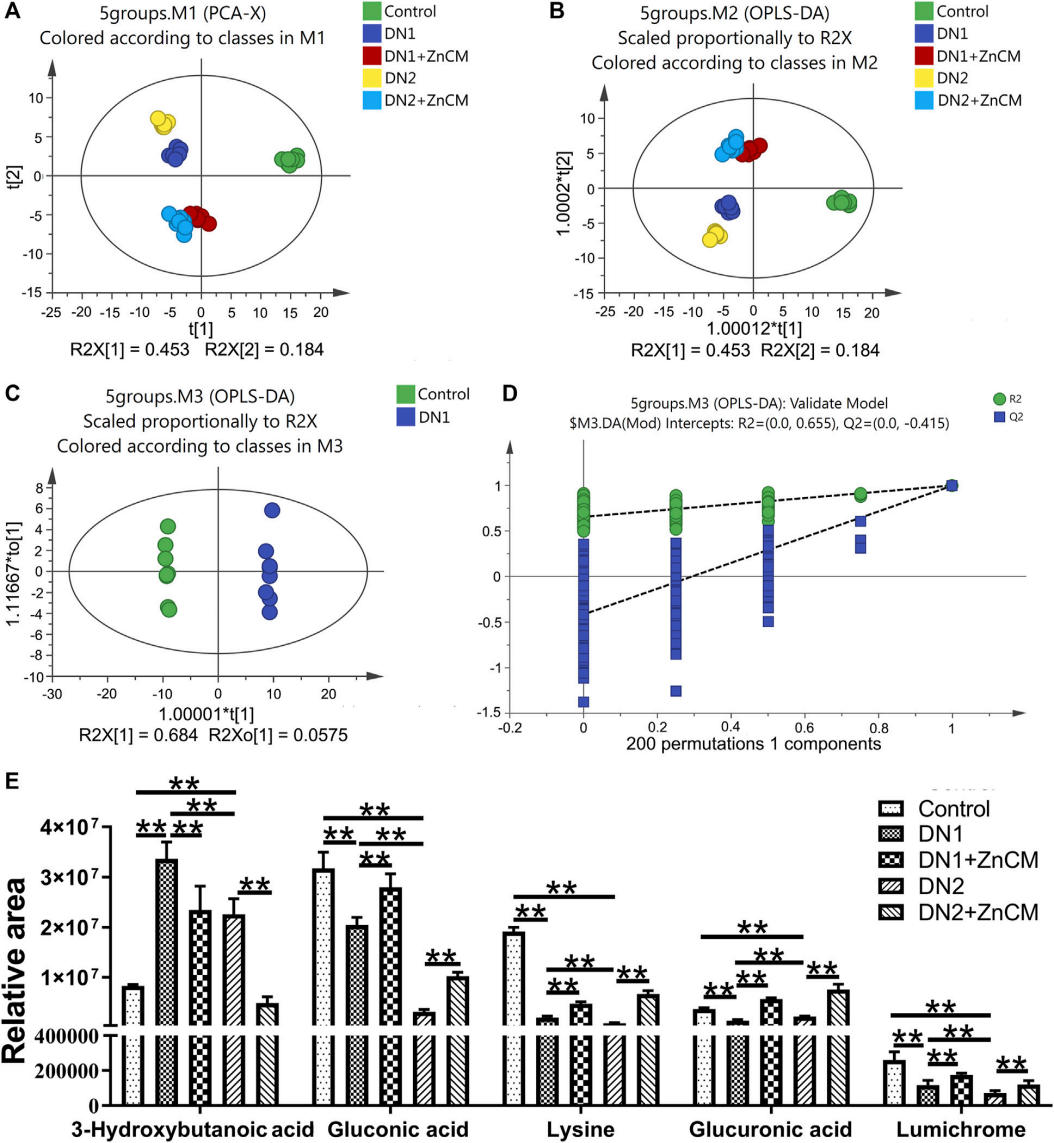
ZnCM altered the metabolite profiling in serum in Cd-induced diabetic nephropathy rats. **(A, B)** The composition of metabolite profiling analyzed by PCA and OPLS-DA. **(C)** OPLS-DA score plot and **(D)** corresponding permutation plot (200 times) between the control rats and the STZ-induced rats obtained from GC–MS. **(E)** The relative intensities of selected metabolites in the serum. Data are shown as means ± SD (*n* = 8) and analyzed by two-way ANOVA followed by Tukey’s multiple comparison tests. *p < 0.05 and **p < 0.01; ns indicates no significance.

Because ZnCM could regulate the gut microbiota composition that affects serum metabolites, we next performed GC–MS to examine the metabolite profiles in the serum of Cd-induced rats with diabetic nephropathy. Principal component analysis (PCA) and orthogonal projection to latent structures-discriminant analysis (OPLS-DA) represent a clear separation of metabolite profiles among different groups, indicating significant changes in metabolites in rats with diabetic nephropathy, which could be remoulded by ZnCM supplementation (Figures 5A, B). The representative total ion chromatograms (TICs) are shown in Supplementary Figure S1. Subsequently, the serum samples were clearly separated into two blocks according to their metabolic profiles of different groups by the score plot of OPLS-DA in both positive and negative ion modes (Supplementary Figure S2). For example, as shown in Figures 5C, D, the results suggested that these metabolites were significantly altered in the control group compared with the STZ-treated group. A total of 135 differential metabolites in the serum were identified in rats with diabetic nephropathy (Supplementary Tables S3–S8). Notably, five differential metabolites, including gluconic acid, lysine, lumichrome, 3-hydroxybutanoic acid, and glucuronic acid, were significantly changed in different groups. Among them, the contents of 3-hydroxybutanoic acid in the serum were clearly increased, and the contents of gluconic acid, lysine, lumichrome, and glucuronic acid were significantly decreased in STZ-treated rats when compared to controls (Figure 5E). Importantly, the serum contents of these five differential metabolites in STZ-treated rats were significantly restored by ZnCM treatment to a certain extent. These findings suggest that oral treatment with ZnCM could reshape the metabolite profiles and improve metabolic disorders in rats with diabetic nephropathy.

### 3.6 Correlations among clinical symptoms, zinc homeostasis, metabolites, and gut microbiota

**FIGURE 6:**
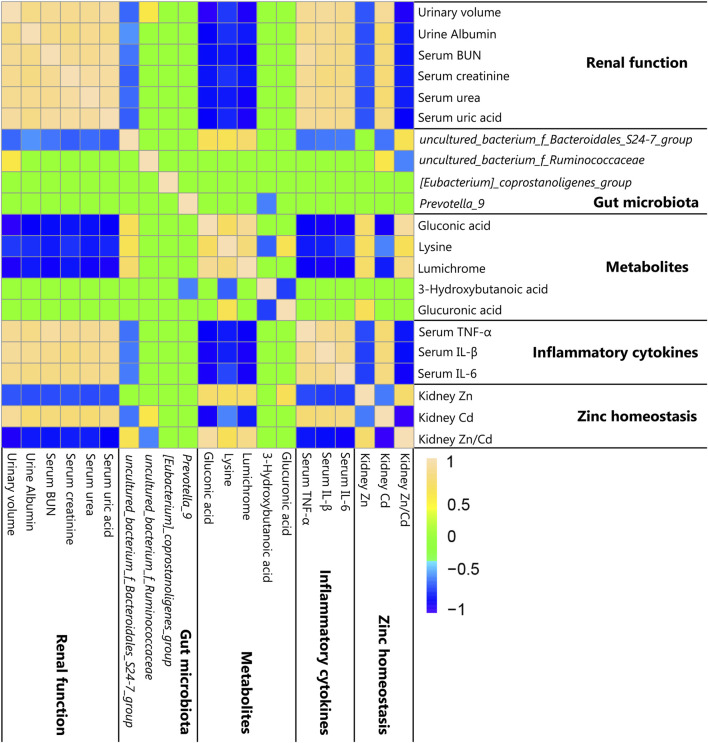
Correlation among clinical symptoms, zinc homeostasis, metabolites, and gut microbiota shown by heat map (*n* = 8). Yellow, green, and blue squares indicate significant positive correlations, non-significant correlations, and significant negative correlations, respectively. Spearman’s and *p* values’ correlation coefficients (|r| > 0.5 and *p* < 0.05) were considered statistically significant.

To explore the relationships among metabolites, cytokines, zinc homeostasis, clinical symptoms, and gut microbiota, Spearman’s correlation analysis was adopted in our study (|r| > 0.5, *p* < 0.05). Spearman’s and *p* values’ correlation coefficient datasets are shown in Supplementary Tables S9, S10. The relative abundance of ZnCM-enriched *uncultured_bacterium_f_Bacteroidales_S24-7_group* (*S24-7_group*) was positively associated with serum levels of gluconic acid, lysine, and lumichrome as well as the renal Zn/Cd ratio, and was negatively correlated with clinical symptoms and serum levels of inflammatory cytokines and renal Cd contents (Figure 6). The relative abundance of ZnCM-decreased *uncultured_bacterium_f_Ruminococcaceae* was found to be negatively correlated with the kidney Zn/Cd ratio and positively correlated with the kidney Cd ratio (Figure 6). Furthermore, the levels of renal Zn and Zn/Cd were positively correlated with metabolites (gluconic acid, lysine, and lumichrome), whereas they were negatively correlated with the serum levels of inflammatory cytokines (Figure 6). Taken together, these findings suggest that the homeostasis of renal zinc and the production of beneficial metabolites such as gluconic acid, lysine, and lumichrome might be at least in part related to the regulation of gut microbiota such as *Bacteroidales_S24-7_group* and *Ruminococcaceae*, which may be in charge of protective effects of ZnCM against the development of diabetic nephropathy in rats exposed by Cd.

### 3.7 Fecal microbial transplantation mitigated renal dysfunction, inflammation, and zinc dyshomeostasis in Cd-exposed rats with diabetic nephropathy

**FIGURE 7:**
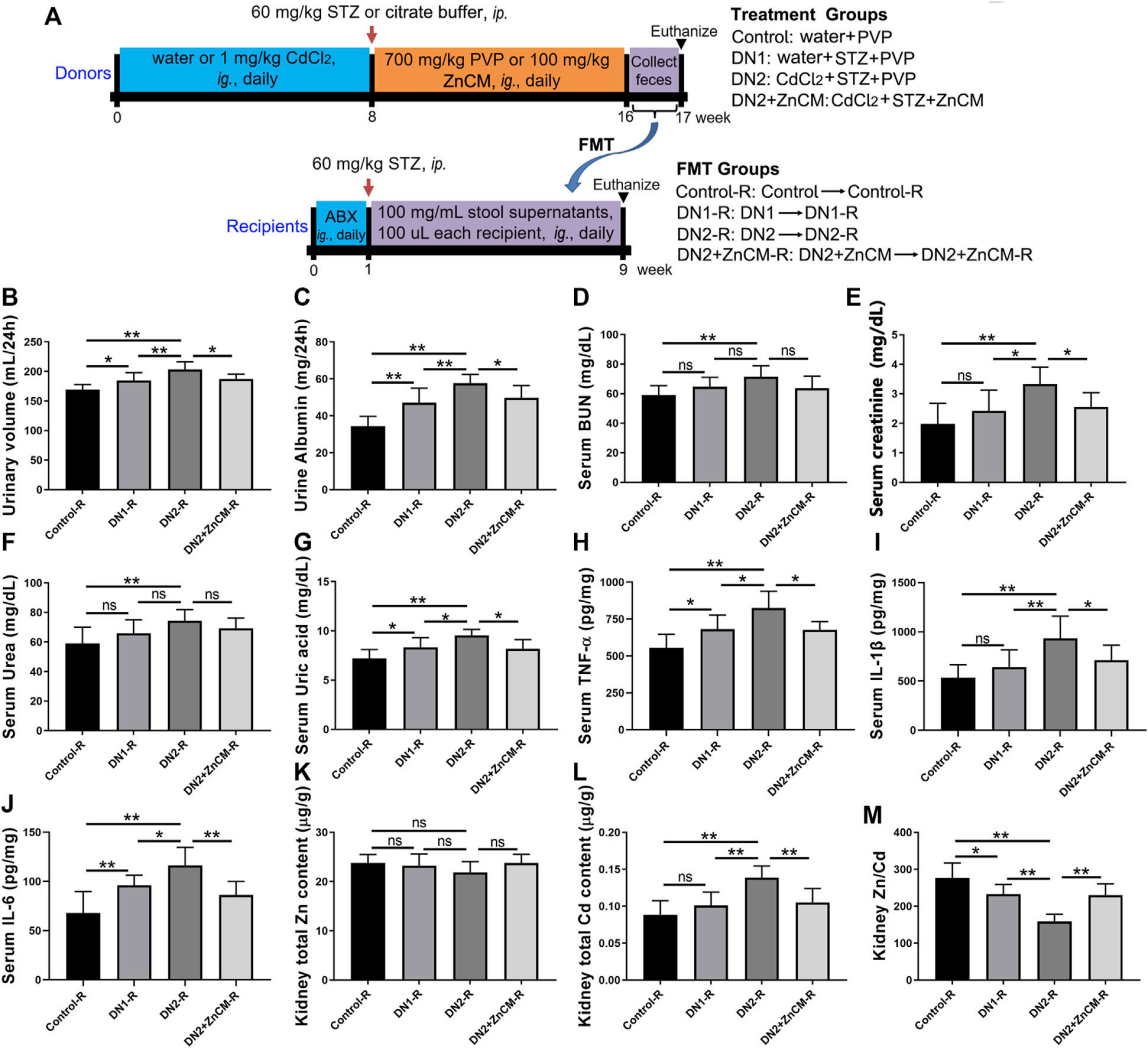
Effects of fecal microbiota transplantation **(**FMT) on renal function, inflammation, and zinc homeostasis in STZ-treated rats exposed to Cd. Recipient rats daily received fecal microbiota from donor rats by oral gavage for 8 weeks. **(A)** The experimental scheme of FMT. Fecal microbiota from control, DN1, DN2, and DN2 + ZnCM rats were transplanted to antibiotics (ABX)-pretreated rats treated by STZ and Cd (*n* = 6). **(B, C)** The levels of urinary volume and urine albumin of recipient rats. **(D–G)** The levels of BUN, creatinine, urea and uric acid in the serum. **(H–J)** The serum levels of inflammatory cytokines TNF-α, IL-1β, and IL-6. **(K–M)** The contents of the Zn, Cd, and Zn/Cd ratio in the kidney. Data are shown as means ± SD (*n* = 6) and analyzed by one-way ANOVA followed by the original FDR method of the Benjamini and Hochberg test. *p < 0.05 and **p < 0.01; ns indicates no significance.

In order to investigate the role of gut microbiota in the mitigation of ZnCM on diabetic nephropathy fecal microbiota from the control, DN1, DN2, and DN2+ZnCM-treated rats were collected and transplanted to the corresponding groups of recipient rats (Figure 7A). Compared to the control-R group, a significant decrease in the renal Zn/Cd ratio and an increase in the urinary volume and the contents of urine albumin and serum creatinine were observed in DN1-R and DN2-R groups. In addition, higher levels of uric acid, TNF-α, IL-1β, and IL-6 in the serum were found in both DN1-R and DN2-R groups than those in the control-R group. Nevertheless, there are no significant differences in the levels of BUN, serum urea, and renal total Zn between the DN1-R group and DN2-R group (Figure 7B-m). Notably, the DN2 + ZnCM-R group not only displayed lower levels of urinary volume and urine albumin but also showed a remarkable decrease in the serum levels of creatinine, uric acid, TNF-α, IL-1β, and IL-6, as well as total Cd and Zn/Cd when compared to the DN2-R group (Figures 7B–M). These results indicate that the ability of ZnCM to regulate zinc homeostasis and exert renal protective effects in Cd-exposed rats with diabetic nephropathy may be mediated by the gut microbiota.

### 3.8 Fecal microbial transplantation reshaped the composition of gut microbiota in diabetic nephropathy rats exposed to Cd

**FIGURE 8:**
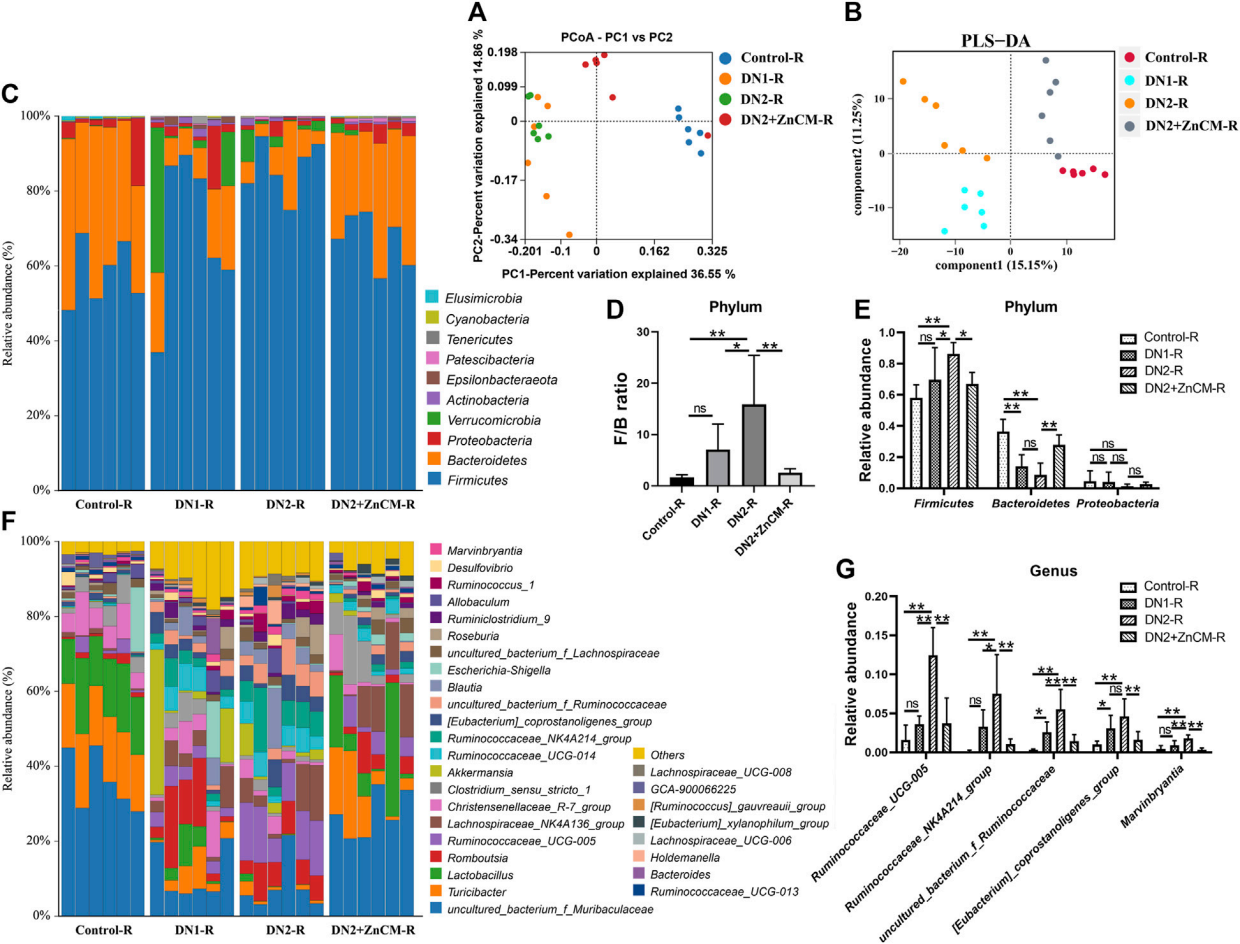
Fecal microbiota transplantation reproduced the ZnCM-induced structure of the gut microbiota in recipient rats. **(A, B)** The composition of the gut microbiota analyzed by PCoA and PLS-DA. **(C)** The relative abundance of the top nine abundant bacteria at the phylum level. **(D)** The ratio of *Firmicute*s/*Bacteroides*. **(E)** The relative abundance of *Firmicutes*, *Bacteroides*, and *Proteobacteria*. **(F)** The relative abundance of top 30 abundant bacteria at the genus level. **(G)** The relative abundance of major altered bacteria at the genus level. Data are shown as means ± SD (*n* = 6) and analyzed by one-way ANOVA followed by the original FDR method of the Benjamini and Hochberg test. *p < 0.05 and **p < 0.01; ns indicates no significance.

To further confirm the ability of ZnCM to modulate the gut microbiota, we analyzed the gut microbiota composition in recipients. PCoA and PLS-DA revealed that the DN2 + ZnCM-R group had a dramatically different microbial landscape from the DN1-R group and DN2-R group (Figures 8A, B). Compared to the DN1-R group, a higher relative abundance of *Firmicutes* and a lower F/B ratio and relative abundance of *Bacteroidetes* were observed in the DN2-R group at the phylum level (Figures 8C–E). The relative abundance of *Ruminococcaceae_UCG-005*, *Ruminococcaceae_NK4A214_group*, *uncultured_bacterium_f_Ruminococcaceae* [*Eubacterium*]*_coprostanoligenes_group*, and *Marvinbryantia* were significantly increased in the DN2-R group at the genus level compared to the DN1-R group (Figures 8F, G). Notably, these changes in the gut microbiota caused by the transplantation of fecal microbiota from Cd-exposed diabetic nephropathy rats were significantly attenuated in the DN2 + ZnCM-R groups (Figures 8F, G). These alterations in the structure and composition of the gut microbiota in the recipient rats were similar to those in the donor rats (Figure 4). These results indicate that gut microbiota may be responsible for the protective effects of ZnCM against diabetic nephropathy.

## 4 Discussion

Acute or chronic Cd exposure has been found to initiate multi-system injuries such as insulin-producing β-cell damage, immune disorder, diabetes, and diabetic retinopathy (El et al., 2012; Filippini et al., 2022). The kidney acts as the major accumulative and vulnerable target organ of Cd exposure, but the unclear mechanism of Cd-aggravated diabetic nephropathy restricts the development of preventive and therapeutic strategies. In the present study, we found that ZnCM treatment effectively improved clinical symptoms, inflammation, and zinc dyshomeostasis in diabetic nephropathy rats exposed to Cd. The administration of ZnCM significantly ameliorated the dysbiosis of fecal microbiota and serum metabolite profiling in Cd-exposed rats with diabetic nephropathy. Transferring of fecal microbiota from ZnCM-treated rats significantly improved clinical symptoms, inflammation, and zinc dyshomeostasis in diabetic nephropathy rats. These findings reveal that the protective effects of ZnCM on diabetic nephropathy may be dependent on the gut microbiota. The ZnCM might be a prospective therapeutic agent for diabetic nephropathy in terms of its strong efficacy.

Diabetic nephropathy is characterized by increased microalbuminuria and macroalbuminuria, and changes in renal morphology such as nodular glomerulosclerosis formation, interstitial fibrosis, and the decrease in glomerular thickness and endothelial cell fenestration (Fineberg et al., 2013). The progression of diabetic nephropathy can be induced by a complex inheritance–environment interaction. For instance, a positive correlation between the environmental pollutant Cd exposure and the incidence and severity of diabetes and its complications has been established in recent epidemiological studies (Edwards and Prozialeck, 2009; Fevrier-Paul et al., 2021). Cd exposure can activate inflammatory response, disturb renin–angiotensin–aldosterone system, and produce mutagenic effect, which may be conducive to the development of diabetes and its complications. Drugs targeted to inflammatory cytokines, adrenocorticotropic hormone receptor (ACTH), TLR4/NF-κB, and TGF-β1 receptors partly prevent the occurrence and development of diabetic nephropathy in clinical trials (Rao et al., 2019). In line with these findings, we found that Cd exposure significantly aggravated clinical symptoms of diabetic nephropathy, renal inflammation, and zinc dyshomeostasis in STZ-treated rats, demonstrating the destructive effect of Cd exposure on renal dysfunction. Cd-induced changes in STZ-treated rats could be improved by ZnCM supplementation. Furthermore, the administration of ZnCM clearly reduces the Cd-loaded local and systemic inflammation that is considered the critical reason for the progression of diabetic nephropathy. Meanwhile, ZnCM inhibited the activation of the inflammation-related TLR4/NF-κB signaling pathway, which is helpful in the alleviation of the progression of diseases (Sun et al., 2021; Sun et al., 2023). The development of inflammation is related to zinc deficiency, which induces a decrease in the innate and adaptive immune response (Bonaventura et al., 2015). Zinc homeostasis is tightly modulated by several transporters and regulators. Changes in zinc status are particularly susceptible to the survival, proliferation, and differentiation of different cells in organs. Fortunately, in addition to the modulation of nephrotoxicity and inflammation of diabetes, ZnCM treatment significantly reduced Cd-induced zinc dyshomeostasis, as indicated by increased renal zinc levels and Zn/Cd ratio and decreased Cd contents in the kidney. Furthermore, ZnCM significantly downregulated the Cd-increased ZIP14, whose high expression increases the accumulation of Cd and leads to zinc deficiency (Min et al., 2013). These profitable effects of ZnCM may be attributed to the success of zinc supplementation because zinc may replace Cd by competitively binding to ZIP14 and alleviating the inflammatory response and zinc dyshomeostasis.

Zinc utilization is vitally regulated by the gut. Zinc deficiency impairs intestinal permeability and induces alterations in the gut microbial ecology (Reed et al., 2015). Gut microbiota dysbiosis has been linked with the process of inflammatory bowel disease, diabetes mellitus, and diabetic complications such as diabetic nephropathy (Zhang et al., 2022). A significant alteration in the composition of the gut microbiota and an increased ratio of *Firmicutes* and *Bacteroidetes* (F/B), the biomarker of gut dysbiosis, were observed in diabetic nephropathy people or animals, which is associated positively with blood glucose concentration (Zhao et al., 2019). Consistent with these findings, we observed that the F/B ratio was noticeably higher in either diabetic nephropathy rats or those exposed to Cd than that of controls. Notably, ZnCM treatment markedly reshaped the composition of the gut microbiota and decreased the F/B ratio in rats with diabetic nephropathy, suggesting that ZnCM effectively maintained or rebalanced intestinal microflora disorder to a healthy status in STZ-treated rats exposed to Cd. In addition, we discovered several useful bacteria, such as *Bacteroidales_S24-7_group*, *[Eubacterium]_Coprostanogenes_group*, and *Prevotella_9*, that were significantly changed after ZnCM administration in diabetic nephropathy rats treated by Cd. Among them, the *Bacteroidales_S24-7_group* has been considered as a gut probiotic that negatively correlated with the lipopolysaccharide, blood glucose, HOMA-IR, and inflammatory response, and positively correlated with the SCFAs content and zinc transporter 2 in mice (Liu et al., 2018; Xiao et al., 2020). Moreover, oral treatment with ZnCM remarkably reduced the relative abundance of pathogenic bacteria such as *Ruminococcaceae* that were significantly enriched in both diabetes mellitus and inflammatory disease animals (Ma et al., 2020; Kunasegaran et al., 2021). In addition, ZnCM significantly decreased the relative abundance of the [*Eubacterium*]*_Coprostanogenes_group*, which is significantly enriched in T2DM patients with diabetes-associated chronic kidney disease patients and associated with dyslipidemia and plasma concentrations of interleukin-12 and miR-142-5p (Huang et al., 2023). These findings suggest that ZnCM improves inflammation, renal dysfunction, and zinc dyshomeostasis, which may be related to the regulation of the gut microbiota in Cd-induced diabetic nephropathy rats.

**FIGURE 9:**
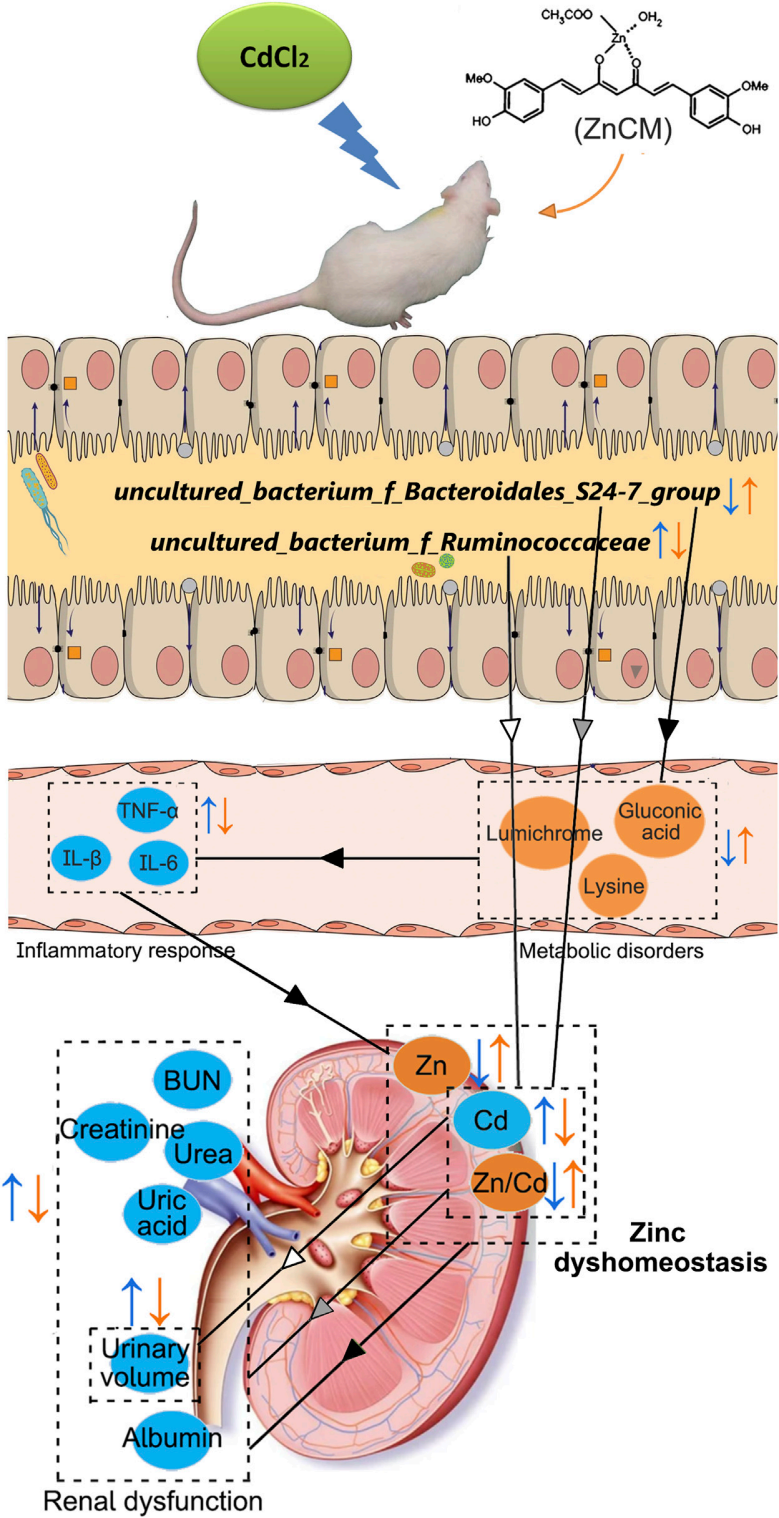
Possible mechanisms involved in the prevention of ZnCM on Cd-aggravated diabetic nephropathy. Oral administration of ZnCM regulated the composition of the gut microbiota and derived metabolites, which are conducive to alleviate inflammatory response and zinc dyshomeostasis, resulting in the mitigation of diabetic nephropathy aggravated by Cd.

Alterations in gut microbiota often affect the host metabolism, especially metabolites that are involved in intestinal barrier function, glucose regulation, energy homeostasis, insulin sensitivity, and inflammation (Tremaroli and Backhed, 2012). In the present study, the serum metabolite profile of control animals is significantly different from that of the rats with diabetic nephropathy or those exposed to Cd. Importantly, oral administration of ZnCM dramatically reshaped the composition of metabolite profiles in diabetic nephropathy rats exposed to Cd. Notably, ZnCM treatment significantly increased five metabolites (gluconic acid, lysine, lumichrome, glucuronic acid, and 3-hydroxybutanoic) that were negatively correlated with clinical phenotypes of diabetic nephropathy and positively correlated with the *Bacteroidales_S24-7_group*. These five metabolites have been documented to exert anti-inflammatory and anti-metabolic disease effects. For example, gluconic acid has been found to inhibit NF-κB activity along with its downstream inflammation signaling cascade (Hsieh et al., 2024). Supplementation with lysine upregulates anti-inflammation cytokines and downregulates pro-inflammation cytokines in fish (Hu et al., 2021). Lumichrome functions as one of the photodegradation products of riboflavin which exerts a critical role in cell oxidation and energy metabolism in rats (Remucal and Mcneill, 2011). These observations together indicate that treatment with ZnCM could improve the metabolites, which are beneficial for the remission of diabetic nephropathy and related to the regulation of the gut microbiota. According to the results of the correlation analysis, the imbalance in gut microbiota, metabolites, cytokines, and zinc homeostasis might be the underlying pathological mechanism of DN injury, and ZnCM might be involved in the regulation of the microbiota–metabolism–inflammation–zinc homeostasis axis and the microbiota–zinc homeostasis axis to ameliorate renal dysfunction in Cd-induced DN (Figure 9). The beneficial effect induced by ZnCM-regulated gut microbiota on the improvement of renal dysfunction, inflammation, and zinc homeostasis was further confirmed by the FMT. Transfering fecal microbiota from diabetic nephropathy rats treated by ZnCM partially recapitulated the composition of the gut microbiota and significantly reduced renal dysfunction, inflammation, and zinc dyshomeostasis in Cd-exposed rats with diabetic nephropathy. These observations further support the concept that the gut bacteria are involved in diabetic nephropathy in Cd-induced rats. However, one of the major open questions is that do fecal suspensions contain negligible amounts of ZnCM, which may directly influence the determination of the effect of the gut microbiota? Further research is needed to explore the role of specific bacteria in the prevention of diabetic nephropathy in the future.

## 5 Conclusion

In conclusion, oral consumption of ZnCM effectively mitigated clinical phenotypes, inflammatory response, and zinc dyshomeostasis in Cd-exposed rats with diabetic nephropathy. Simultaneously, ZnCM treatment not only reversed the composition of fecal microbiota but also changed the serum metabolite profiling in diabetic nephropathy rats exposed to Cd. Fecal microbiota from rats orally treated by ZnCM could reduce clinical phenotypes and zinc dyshomeostasis in Cd-exposed rats with diabetic nephropathy, suggesting that the prevention of ZnCM on Cd-aggravated diabetic nephropathy was partly dependent on the gut microbiota. These findings indicate that ZnCM supplementation may be a promising candidate to mitigate diabetic nephropathy. However, more detailed mechanisms are needed to be investigated in further studies.

## Data Availability

The data presented in the study are deposited in the NCBI Sequence Read Archive repository, accession numbers PRJNA864569 and PRJNA1015156.

## Abbreviations

BUN, blood urea nitrogen; Cd, cadmium; CM, curcumin; DN, diabetic nephropathy; FMT, fecal microbiota transplantation; Met, metformin; NF-κB p65, nuclear factor-κB p65; PVP-30, polyvinylpyrrolidone K30; SD, standard deviation; STZ, streptozotocin; Scr, serum creatinine; TLR4, toll-like receptor 4; ZIP14, Zrt, Irt-related protein 14; Zn, zinc; ZnCM, Zn(II)–curcumin.
