# Supplementary material for: Zn(II)–curcumin prevents cadmium-aggravated diabetic nephropathy by regulating gut microbiota and zinc homeostasis
Source: Front Pharmacol. 2024 Jun 5;15:1411230. doi: 10.3389/fphar.2024.1411230 (PMC11188322; doi:10.3389/fphar.2024.1411230)
Supplement: Supplementary file 1 [file Table1.docx]

**Supplementary Tables**

**Table S1. The primary and secondary antibodies**

| NF-κBp-p65 | #3033; Cell Signaling Technology, Inc., Danvers, MA, USA |
| --- | --- |
| NF-κBp65 | #6956; Cell Signaling Technology, Inc., Danvers, MA, USA |
| TLR4 | AF8187; Beyotime, Shanghai, China |
| MYD88 | AF7524; Beyotime, Shanghai, China |
| IκBα | AI096; Beyotime, Shanghai, China |
| p-IκBα | E-AB-20911; Elabscience Biotechnology Co., Ltd, Wuhan, China |
| SLC39A14 [ZIP14] | 25236; SAB, California, USA |
| β-actin | 20536-1-AP; Proteintech Group, Inc., Wuhan, China |

**Table S2. GC-MS instrument settings**

| Column | DBS-MS column (30m × 250 μm i.d. × 0.25 μm, Agilent J & W Scientific, Folsom, CA, USA) |
| --- | --- |
| Initial temp. (℃) | 85 |
| Temp. ascending speed (℃/min) | 15 |
| Termination temp. (℃) | 270 |
| Injection mode | Splitless |
| Injection Vol. (μL) | 0.5 |
| Inlet and interface temp. (℃) | 270 |
| EI temp. (℃) | 230 |
| Ionization voltage (eV) | 70 |
| Quadrupole temp. (℃) | 150 |
| Carrier gas | Helium (≥99%) |
| Flow rate (mL/min) | 1.0 |
| Scanning mode (m/z) | 50-600 |

**Table S3. Peaks identified in TIC chromatogram of serum from five groups after peak alignment.**

| **No.** | **metabolites** | **Retention Time** |
| --- | --- | --- |
| 1 | Cysteamine | 5.2646 |
| 2 | Lactic acid | 6.0801 |
| 3 | Hexanoic acid | 6.4823 |
| 4 | Alanine | 7.0822 |
| 5 | 2-hydroxy-Butanoic acid | 7.6021 |
| 6 | Leucine | 8.3052 |
| 7 | 3-hydroxy-Butanoic acid | 8.4087 |
| 8 | Butanoic acid | 8.692 |
| 9 | Valine | 9.783 |
| 10 | Sulfuric acid | 9.8851 |
| 11 | Benzoic acid | 10.5995 |
| 12 | Urea | 10.9417 |
| 13 | Norleucine | 11.2628 |
| 14 | Glycerol | 11.3561 |
| 15 | Phosphoric acid | 11.4372 |
| 16 | Isoleucine | 11.8116 |
| 17 | Proline | 11.8838 |
| 18 | Glycine | 12.1171 |
| 19 | Isobutanoic acid | 12.4471 |
| 20 | Lumichrome | 12.6336 |
| 21 | Glyceric acid | 12.7937 |
| 22 | Uracil | 12.9204 |
| 23 | Fumaric acid | 13.337 |
| 24 | Serine | 13.5825 |
| 25 | Threonine | 14.228 |
| 26 | Thymine | 14.5257 |
| 27 | Hydantoin | 14.5634 |
| 28 | Malonic acid | 14.5879 |
| 29 | Sphingosine | 14.609 |
| 30 | Putrescine | 14.8011 |
| 31 | 2,4-dihydroxy-Butanoic acid | 14.9068 |
| 32 | beta-Alanine | 15.2545 |
| 33 | Ethanolamine | 15.2723 |
| 34 | 5-hydroxy-Tryptophan | 15.8422 |
| 35 | 5-hydroxy-Lysine | 15.9021 |
| 36 | Malic acid | 16.3209 |
| 37 | Dodecanol | 17.0387 |
| 38 | Threitol | 17.3153 |
| 39 | Methionine | 17.482 |
| 40 | 1-Pyrroline-3-hydroxy-5-carboxylic acid | 17.5053 |
| 41 | Cytosine | 17.5275 |
| 42 | Aspartic acid | 17.572 |
| 43 | 4-hydroxy-Proline | 17.6785 |
| 44 | 3-hydroxy-Proline | 17.6853 |
| 45 | Ditertbutylphenol | 17.9497 |
| 46 | Erythronic acid | 18.1308 |
| 47 | Creatinine | 18.2441 |
| 48 | Cysteine | 18.3597 |
| 49 | 2-hydroxy-Glutaric acid | 18.894 |
| 50 | Hypotaurine | 19.3195 |
| 51 | Calystegine B2 | 19.4006 |
| 52 | Pyruvic acid | 19.7539 |
| 53 | Glutamic acid | 19.9217 |
| 54 | Phenylalanine | 19.9495 |
| 55 | 2-Imidazolidone-4-carboxylic acid | 20.0606 |
| 56 | 4-hydroxyphenylacetic acid | 20.2816 |
| 57 | n-Tridecan-1-ol | 20.6416 |
| 58 | Dodecanoic acid | 20.6538 |
| 59 | Taurine | 20.8493 |
| 60 | Asparagine | 20.9993 |
| 61 | Lyxose | 21.2382 |
| 62 | Xylulose | 21.4893 |
| 63 | 3-hydroxy-Indole | 21.7203 |
| 64 | Arabitol | 22.1736 |
| 65 | Rhamnose | 22.4847 |
| 66 | n-Eicosan-1-ol | 22.8735 |
| 67 | Carbodiimide | 23.038 |
| 68 | Glycerol-3-phosphate | 23.0691 |
| 69 | Glutamine | 23.2824 |
| 70 | Galactose | 23.4467 |
| 71 | Methionine sulfoxide | 23.4823 |
| 72 | Fructose | 23.9545 |
| 73 | Ornithine | 24.1811 |
| 74 | Pyrophosphate | 24.2146 |
| 75 | Citric acid | 24.2534 |
| 76 | Arginine | 24.3611 |
| 77 | Pinitol | 24.45 |
| 78 | Hippuric acid | 24.6878 |
| 79 | Altrose | 24.7266 |
| 80 | Xylitol | 24.851 |
| 81 | Sorbose | 25.2731 |
| 82 | Allantoin | 25.5221 |
| 83 | Allose | 25.6119 |
| 84 | Idose | 25.922 |
| 85 | Glucose | 26.1831 |
| 86 | Mannose | 26.3619 |
| 87 | Lysine | 26.402 |
| 88 | Gulose | 26.4353 |
| 89 | Hexadecanoic acid | 26.4786 |
| 90 | Glucuronic acid | 26.5886 |
| 91 | Mannitol | 26.6286 |
| 92 | Tyrosine | 26.6519 |
| 93 | Sorbitol | 26.7152 |
| 94 | Galacturonic acid | 26.8619 |
| 95 | Inositol | 26.9741 |
| 96 | Gulonic acid | 27.1274 |
| 97 | 4-hydroxy-Quinazoline | 27.173 |
| 98 | Pantothenic acid | 27.6396 |
| 99 | beta-Allose | 27.764 |
| 100 | Lyxonic acid | 28.0839 |
| 101 | Ribonic acid | 28.324 |
| 102 | myo-Inositol | 29.4994 |
| 103 | Octadecadienoic acid | 29.5782 |
| 104 | Uric acid | 29.696 |
| 105 | Octadecenoic acid | 29.7127 |
| 106 | Octadecanoic acid | 30.2137 |
| 107 | Histidine | 30.4725 |
| 108 | Heptadecanoic acid | 30.527 |
| 109 | n-Octadecan-1-ol | 30.527 |
| 110 | Indole-3-lactic acid | 30.8836 |
| 111 | Tryptophan | 31.3647 |
| 112 | 9,12-Octadecadienoic acid | 31.6802 |
| 113 | 9-Octadecenoic acid | 31.7969 |
| 114 | Glucoheptonic acid | 31.9202 |
| 115 | beta-Homoserine | 32.8278 |
| 116 | Gluconic acid | 32.9667 |
| 117 | Glucose-6-phosphate | 33.5366 |
| 118 | Eicosatetraenoic acid | 34.2799 |
| 119 | myo-Inositol-2-phosphate | 35.0187 |
| 120 | n-Heneicosan-1-ol | 35.5329 |
| 121 | Eicosanoic acid | 35.5353 |
| 122 | Xylobiose | 38.4626 |
| 123 | Kestose | 38.5471 |
| 124 | Xanthosine | 39.0815 |
| 125 | Nigerose | 39.9802 |
| 126 | Cellotriose | 40.389 |
| 127 | Gentiobiose | 40.3902 |
| 128 | Trehalose | 40.518 |
| 129 | Sophorose | 40.749 |
| 130 | Glyceraldehyde-3-phosphate | 43.9398 |
| 131 | alpha-Tocopherol | 44.7574 |
| 132 | Cholesterol | 44.8219 |
| 133 | Glycolic acid-2-phosphate | 45.6695 |
| 134 | Campesterol | 45.7917 |
| 135 | beta-Sitosterol | 46.6939 |

**Table S4. Differential metabolites in response to DN1 group vs. Control group.**

| No | Metabolite | VIP | t-test P | FC | Trend |
| --- | --- | --- | --- | --- | --- |
| 1 | Lysine | 1.21661 | <0.000000000000001 | 0.10297 | Down |
| 2 | Urea | 1.21582 | <0.000000000000001 | 0.080762 | Down |
| 3 | Cytosine | 1.21398 | <0.000000000000001 | 0.29876 | Down |
| 4 | Mannose | 1.21261 | 7E-15 | 4.108 | Up |
| 5 | Histidine | 1.21256 | 9E-15 | 7.4625 | Up |
| 6 | Threonine | 1.21175 | 9E-15 | 0.46479 | Down |
| 7 | Ethanolamine | 1.21155 | 1.6E-14 | 6.5852 | Up |
| 8 | Glycerol-3-phosphate | 1.21154 | 1.2E-14 | 0.38611 | Down |
| 9 | Glutamine | 1.2112 | 1.3E-14 | 0.079304 | Down |
| 10 | beta-Homoserine | 1.21008 | 3E-14 | 0.39543 | Down |
| 11 | Cholesterol | 1.20827 | 1.83E-13 | 2.9137 | Up |
| 12 | Gulose | 1.2082 | 1.06E-13 | 117.97 | Up |
| 13 | Tryptophan | 1.20788 | 1.39E-13 | 0.23752 | Down |
| 14 | Glycolic acid-2-phosphate | 1.20718 | 3.06E-13 | 0.23227 | Down |
| 15 | Malic acid | 1.20678 | 5.99E-13 | 0.25781 | Down |
| 16 | Sorbitol | 1.20634 | 4.28E-13 | 66.781 | Up |
| 17 | beta-Alanine | 1.20602 | 7.22E-13 | 0.40098 | Down |
| 18 | 2,4-dihydroxy-Butanoic acid | 1.20558 | 7.14E-13 | 3.5952 | Up |
| 19 | Glucuronic acid | 1.20324 | 1.864E-12 | 0.3721 | Down |
| 20 | Nigerose | 1.20196 | 9.685E-12 | 10.146 | Up |
| 21 | Octadecenoic acid | 1.20125 | 1.959E-12 | 0.39237 | Down |
| 22 | Glucose | 1.20089 | 7.241E-12 | 9.6325 | Up |
| 23 | Ditertbutylphenol | 1.20087 | 3.163E-12 | 0.56858 | Down |
| 24 | 3-hydroxy-Butanoic acid | 1.1988 | 5.312E-12 | 4.0336 | Up |
| 25 | Glucose-6-phosphate | 1.19846 | 2.797E-12 | 0.52997 | Down |
| 26 | myo-Inositol | 1.19774 | 1.4947E-11 | 0.5086 | Down |
| 27 | Fumaric acid | 1.1976 | 2.0425E-11 | 0.30961 | Down |
| 28 | Uric acid | 1.19683 | 1.6029E-11 | 0.52675 | Down |
| 29 | Taurine | 1.19621 | 4.9609E-11 | 0.43473 | Down |
| 30 | Methionine | 1.19569 | 3.2904E-11 | 0.47543 | Down |
| 31 | Allantoin | 1.19554 | 3.457E-11 | 0.27665 | Down |
| 32 | Asparagine | 1.19402 | 3.1053E-11 | 0.3894 | Down |
| 33 | Hypotaurine | 1.1939 | 6.2195E-11 | 0.32294 | Down |
| 34 | Serine | 1.19211 | 7.3915E-11 | 0.60647 | Down |
| 35 | Calystegine B2 | 1.19184 | 9.3765E-11 | 5.0054 | Up |
| 36 | Glyceraldehyde-3-phosphate | 1.1907 | 2.54191E-10 | 0.33261 | Down |
| 37 | Octadecanoic acid | 1.19062 | 1.00325E-10 | 0.51591 | Down |
| 38 | 9,12-Octadecadienoic acid | 1.18758 | 1.38053E-10 | 0.647 | Down |
| 39 | n-Octadecan-1-ol | 1.18736 | 3.71473E-10 | 0.65462 | Down |
| 40 | Creatinine | 1.18626 | 2.2465E-10 | 0.29146 | Down |
| 41 | Benzoic acid | 1.18517 | 2.9643E-10 | 0.39755 | Down |
| 42 | Xylitol | 1.18396 | 4.71162E-10 | 0.69604 | Down |
| 43 | Ornithine | 1.18395 | 4.64701E-10 | 0.29452 | Down |
| 44 | Isobutanoic acid | 1.1839 | 3.54832E-10 | 0.42498 | Down |
| 45 | Pinitol | 1.18 | 9.47677E-10 | 4.6709 | Up |
| 46 | n-Eicosan-1-ol | 1.17979 | 1.55457E-09 | 0.072813 | Down |
| 47 | 3-hydroxy-Indole | 1.17809 | 1.05266E-09 | 2.0688 | Up |
| 48 | Xylobiose | 1.17704 | 6.70772E-10 | 0.36421 | Down |
| 49 | 4-hydroxy-Proline | 1.1768 | 1.41981E-09 | 0.55866 | Down |
| 50 | Octadecadienoic acid | 1.1764 | 1.43243E-09 | 0.57817 | Down |
| 51 | 3-hydroxy-Proline | 1.17367 | 3.48749E-09 | 0.50386 | Down |
| 52 | 2-Imidazolidone-4-carboxylic acid | 1.17347 | 2.79627E-09 | 0.49917 | Down |
| 53 | 9-Octadecenoic acid | 1.17246 | 5.99464E-09 | 0.17203 | Down |
| 54 | Alanine | 1.17218 | 3.74585E-09 | 0.50858 | Down |
| 55 | Inositol | 1.16854 | 8.18628E-09 | 0.61184 | Down |
| 56 | Thymine | 1.16782 | 4.86631E-09 | 0.2581 | Down |
| 57 | 5-hydroxy-Lysine | 1.16636 | 9.20662E-09 | 0.46397 | Down |
| 58 | Galacturonic acid | 1.16605 | 4.90115E-09 | 0.45089 | Down |
| 59 | Hippuric acid | 1.16568 | 1.08517E-08 | 1.6386 | Up |
| 60 | Allose | 1.16441 | 4.04237E-09 | 0.36728 | Down |
| 61 | Dodecanoic acid | 1.15713 | 2.42693E-08 | 0.36701 | Down |
| 62 | Trehalose | 1.1571 | 3.39742E-08 | 5.2951 | Up |
| 63 | Tyrosine | 1.15566 | 4.09448E-08 | 0.47788 | Down |
| 64 | Sphingosine | 1.1544 | 2.1447E-08 | 0.50661 | Down |
| 65 | myo-Inositol-2-phosphate | 1.14988 | 3.21373E-08 | 0.36617 | Down |
| 66 | Aspartic acid | 1.14478 | 7.70823E-08 | 1.5816 | Up |
| 67 | n-Tridecan-1-ol | 1.14188 | 1.24895E-07 | 0.46106 | Down |
| 68 | Hexadecanoic acid | 1.1417 | 1.47301E-07 | 0.36847 | Down |
| 69 | Glucoheptonic acid | 1.12853 | 3.23727E-07 | 0.44327 | Down |
| 70 | Gluconic acid | 1.12764 | 3.59833E-07 | 0.64522 | Down |
| 71 | Methionine sulfoxide | 1.12552 | 6.14471E-07 | 1.6912 | Up |
| 72 | Norleucine | 1.12037 | 5.27848E-07 | 0.52439 | Down |
| 73 | Mannitol | 1.11337 | 8.72303E-07 | 3.2643 | Up |
| 74 | Gulonic acid | 1.11055 | 9.72953E-07 | 0.46558 | Down |
| 75 | Malonic acid | 1.10808 | 8.8756E-07 | 0.43396 | Down |
| 76 | Gentiobiose | 1.09444 | 3.28188E-06 | 4.2436 | Up |
| 77 | 2-hydroxy-Butanoic acid | 1.09304 | 2.80105E-06 | 0.68627 | Down |
| 78 | Eicosatetraenoic acid | 1.0928 | 2.07849E-06 | 0.45371 | Down |
| 79 | Glutamic acid | 1.09241 | 2.07684E-06 | 0.48727 | Down |
| 80 | Lumichrome | 1.08489 | 2.9352E-06 | 0.44567 | Down |
| 81 | Phosphoric acid | 1.07079 | 6.53169E-06 | 0.65488 | Down |
| 82 | Glycine | 1.00166 | 0.00011766 | 0.43016 | Down |

**Table S5. Differential metabolites in response to DN1+ZnCM group vs. DN1 group**

| No | Metabolite | VIP | T-test P | FC | Trend |
| --- | --- | --- | --- | --- | --- |
| 1 | 4-hydroxyphenylacetic acid | 1.32721 | 1.3E-14 | 6.0196 | Up |
| 2 | Glucuronic acid | 1.32545 | <0.000000000000001 | 4.1391 | Up |
| 3 | n-Eicosan-1-ol | 1.32521 | 3.969E-12 | 3.769 | Up |
| 4 | Sorbitol | 1.32379 | 4.82E-13 | 0.015746 | Down |
| 5 | Cysteine | 1.31644 | 1.3475E-11 | 2.3516 | Up |
| 6 | Glucose | 1.31598 | 1.07959E-10 | 0.21302 | Down |
| 7 | 3-hydroxy-Indole | 1.31284 | 1.0511E-11 | 2.369 | Up |
| 8 | Malic acid | 1.30662 | 1.04434E-10 | 2.4615 | Up |
| 9 | Aspartic acid | 1.30651 | 8.1174E-11 | 1.8536 | Up |
| 10 | Erythronic acid | 1.30598 | 8.0892E-11 | 1.6769 | Up |
| 11 | Lysine | 1.30015 | 6.6446E-11 | 2.4203 | Up |
| 12 | Taurine | 1.29948 | 1.05079E-09 | 2.0563 | Up |
| 13 | Uracil | 1.29849 | 2.26017E-09 | 2.2571 | Up |
| 14 | Mannitol | 1.29392 | 2.17173E-09 | 2.2464 | Up |
| 15 | Lyxose | 1.28827 | 8.45001E-10 | 0.45944 | Down |
| 16 | Octadecadienoic acid | 1.28604 | 1.71264E-09 | 0.44023 | Down |
| 17 | beta-Alanine | 1.28562 | 3.67918E-09 | 1.9959 | Up |
| 18 | Hydantoin | 1.28292 | 6.48291E-09 | 1.4748 | Up |
| 19 | Galacturonic acid | 1.28089 | 3.65993E-09 | 2.6435 | Up |
| 20 | Pinitol | 1.28013 | 4.48723E-09 | 0.23197 | Down |
| 21 | Xanthosine | 1.27735 | 1.17741E-08 | 0.47594 | Down |
| 22 | beta-Homoserine | 1.27721 | 7.51984E-09 | 1.5571 | Up |
| 23 | Threitol | 1.26904 | 5.86629E-09 | 2.3517 | Up |
| 24 | Uric acid | 1.26878 | 2.75624E-08 | 1.5054 | Up |
| 25 | Threonine | 1.26851 | 1.69616E-08 | 1.4554 | Up |
| 26 | Cholesterol | 1.26101 | 5.24747E-08 | 0.68944 | Down |
| 27 | 9-Octadecenoic acid | 1.25843 | 6.52877E-08 | 2.0857 | Up |
| 28 | Gulose | 1.25744 | 9.04218E-08 | 1.7953 | Up |
| 29 | Alanine | 1.25192 | 1.04947E-07 | 1.7361 | Up |
| 30 | Tyrosine | 1.24973 | 1.32414E-07 | 1.4433 | Up |
| 31 | Glycolic acid-2-phosphate | 1.24964 | 1.9798E-07 | 1.7163 | Up |
| 32 | Glycerol | 1.24041 | 4.13388E-07 | 0.35501 | Down |
| 33 | Butanoic acid | 1.23563 | 6.97198E-07 | 2.2987 | Up |
| 34 | Altrose | 1.2352 | 4.49464E-08 | 1.7845 | Up |
| 35 | 2-Imidazolidone-4-carboxylic acid | 1.22358 | 1.16564E-06 | 1.5236 | Up |
| 36 | Methionine | 1.2206 | 9.03229E-07 | 1.4295 | Up |
| 37 | Kestose | 1.22032 | 1.03722E-06 | 1.7709 | Up |
| 38 | Ribonic acid | 1.20733 | 1.1335E-06 | 0.71511 | Down |
| 39 | Allantoin | 1.20383 | 1.35128E-06 | 3.9797 | Up |
| 40 | Octadecenoic acid | 1.20283 | 1.41482E-06 | 1.3778 | Up |
| 41 | 5-hydroxy-Tryptophan | 1.20257 | 1.94933E-06 | 1.9442 | Up |
| 42 | Fumaric acid | 1.19931 | 5.16881E-06 | 1.567 | Up |
| 43 | myo-Inositol | 1.195 | 2.6482E-06 | 1.3953 | Up |
| 44 | Phenylalanine | 1.18811 | 8.68736E-06 | 1.4156 | Up |
| 45 | Gulonic acid | 1.18256 | 5.32823E-06 | 1.5666 | Up |
| 46 | Heptadecanoic acid | 1.1802 | 6.20066E-06 | 0.7167 | Down |
| 47 | Gluconic acid | 1.17524 | 7.26454E-06 | 1.3675 | Up |
| 48 | Rhamnose | 1.1748 | 9.67021E-06 | 19.291 | Up |
| 49 | Asparagine | 1.14949 | 2.3237E-05 | 1.4659 | Up |
| 50 | alpha-Tocopherol | 1.14807 | 1.55381E-05 | 2.2042 | Up |
| 51 | Methionine sulfoxide | 1.12698 | 4.79874E-05 | 0.69829 | Down |
| 52 | Leucine | 1.11972 | 7.91724E-05 | 0.60893 | Down |
| 53 | Lumichrome | 1.11216 | 5.81115E-05 | 1.5065 | Up |
| 54 | Ornithine | 1.11206 | 6.65759E-05 | 1.4767 | Up |
| 55 | Creatinine | 1.09322 | 0.000153234 | 1.5164 | Up |
| 56 | n-Tridecan-1-ol | 1.08883 | 9.61142E-05 | 1.3905 | Up |
| 57 | Glutamic acid | 1.08785 | 0.00011743 | 1.7456 | Up |
| 58 | Eicosanoic acid | 1.08601 | 0.000251525 | 0.63418 | Down |
| 59 | Histidine | 1.07472 | 0.000364057 | 0.65984 | Down |
| 60 | Eicosatetraenoic acid | 1.07003 | 0.000258152 | 1.8093 | Up |
| 61 | 3-hydroxy-Butanoic acid | 1.06575 | 0.000218845 | 0.69837 | Down |
| 62 | Glyceraldehyde-3-phosphate | 1.05754 | 0.000518146 | 1.6123 | Up |
| 63 | Malonic acid | 1.0425 | 0.000250563 | 1.438 | Up |
| 64 | Glutamine | 1.03978 | 0.000584392 | 2.037 | Up |

**Table S6. Differential metabolites in response to DN2 group vs. Control group.**

| No | Metabolite | VIP | t-test P | FC | Trend |
| --- | --- | --- | --- | --- | --- |
| 1 | Lysine | 1.16245 | <0.000000000000001 | 0.04467 | Down |
| 2 | Sophorose | 1.16229 | <0.000000000000001 | 10.385 | Up |
| 3 | Cytosine | 1.16115 | <0.000000000000001 | 0.28808 | Down |
| 4 | Mannose | 1.16099 | <0.000000000000001 | 4.1375 | Up |
| 5 | Glycerol-3-phosphate | 1.16089 | <0.000000000000001 | 0.30284 | Down |
| 6 | Cholesterol | 1.16086 | <0.000000000000001 | 3.7515 | Up |
| 7 | beta-Homoserine | 1.15982 | <0.000000000000001 | 0.15398 | Down |
| 8 | Hexadecanoic acid | 1.15981 | <0.000000000000001 | 0.46254 | Down |
| 9 | Ethanolamine | 1.15939 | <0.000000000000001 | 6.5379 | Up |
| 10 | Threonine | 1.15927 | 3E-15 | 0.53384 | Down |
| 11 | Glutamine | 1.15863 | <0.000000000000001 | 0.093447 | Down |
| 12 | Urea | 1.15817 | 5E-15 | 0.075072 | Down |
| 13 | Sorbitol | 1.15715 | 7E-15 | 141.79 | Up |
| 14 | n-Octadecan-1-ol | 1.15638 | 4.4E-14 | 0.29275 | Down |
| 15 | Ditertbutylphenol | 1.15636 | 7.6E-14 | 0.54989 | Down |
| 16 | Erythronic acid | 1.1559 | 6.6E-14 | 0.49185 | Down |
| 17 | 3-hydroxy-Indole | 1.15527 | 4.7E-14 | 5.0892 | Up |
| 18 | Trehalose | 1.15479 | 1.31E-13 | 14.516 | Up |
| 19 | Pinitol | 1.15338 | 1.41E-13 | 5.2874 | Up |
| 20 | beta-Alanine | 1.15309 | 8.45E-13 | 0.39346 | Down |
| 21 | Glycolic acid-2-phosphate | 1.15223 | 8.74E-13 | 0.28864 | Down |
| 22 | Gluconic acid | 1.15193 | 5.24E-13 | 0.09884 | Down |
| 23 | Hypotaurine | 1.15171 | 2.308E-12 | 0.17198 | Down |
| 24 | Methionine sulfoxide | 1.15166 | 2.36E-13 | 2.014 | Up |
| 25 | Glucose | 1.15113 | 2.8E-13 | 10.338 | Up |
| 26 | Taurine | 1.15092 | 5.31E-13 | 0.44671 | Down |
| 27 | Serine | 1.15017 | 1.83E-12 | 0.6086 | Down |
| 28 | Galacturonic acid | 1.14901 | 2.888E-12 | 0.59189 | Down |
| 29 | Octadecenoic acid | 1.14868 | 8.15E-13 | 0.43293 | Down |
| 30 | Glyceric acid | 1.14747 | 4.862E-12 | 0.61048 | Down |
| 31 | Alanine | 1.14709 | 6.002E-12 | 0.55659 | Down |
| 32 | Benzoic acid | 1.1467 | 2.225E-12 | 0.24359 | Down |
| 33 | Aspartic acid | 1.14649 | 1.509E-12 | 1.8974 | Up |
| 34 | Tryptophan | 1.14603 | 3.218E-12 | 0.40173 | Down |
| 35 | Glyceraldehyde-3-phosphate | 1.14565 | 9.905E-12 | 0.35423 | Down |
| 36 | Asparagine | 1.1456 | 5.577E-12 | 0.50221 | Down |
| 37 | 2,4-dihydroxy-Butanoic acid | 1.14528 | 4.235E-12 | 4.1567 | Up |
| 38 | Heptadecanoic acid | 1.14515 | 6.787E-12 | 0.50355 | Down |
| 39 | Sulfuric acid | 1.14105 | 3.9683E-11 | 25.436 | Up |
| 40 | Glucose-6-phosphate | 1.1403 | 3.1007E-11 | 0.6802 | Down |
| 41 | Glucuronic acid | 1.14009 | 6.5226E-11 | 0.58956 | Down |
| 42 | Ornithine | 1.13895 | 7.2585E-11 | 0.26274 | Down |
| 43 | Creatinine | 1.13818 | 6.2546E-11 | 0.32992 | Down |
| 44 | Calystegine B2 | 1.1374 | 5.5099E-11 | 5.0519 | Up |
| 45 | Hippuric acid | 1.13724 | 7.4667E-11 | 1.9173 | Up |
| 46 | Octadecadienoic acid | 1.13573 | 1.12269E-10 | 0.63809 | Down |
| 47 | Malonic acid | 1.13458 | 1.8098E-10 | 0.16067 | Down |
| 48 | Carbodiimide | 1.13441 | 1.09814E-10 | 0.30685 | Down |
| 49 | Allantoin | 1.13424 | 2.74679E-10 | 0.15689 | Down |
| 50 | Cellotriose | 1.13406 | 2.08111E-10 | 13.788 | Up |
| 51 | Fumaric acid | 1.1323 | 8.08493E-10 | 0.40472 | Down |
| 52 | Isobutanoic acid | 1.13157 | 6.08345E-10 | 0.25165 | Down |
| 53 | Gulose | 1.13138 | 3.67501E-10 | 88.886 | Up |
| 54 | Thymine | 1.13131 | 3.4468E-10 | 0.15275 | Down |
| 55 | Glucoheptonic acid | 1.13096 | 4.03937E-10 | 0.088109 | Down |
| 56 | Nigerose | 1.13077 | 4.68599E-10 | 14.62 | Up |
| 57 | Phosphoric acid | 1.12973 | 4.92944E-10 | 0.52644 | Down |
| 58 | n-Eicosan-1-ol | 1.12961 | 1.33018E-09 | 0.063716 | Down |
| 59 | 2-hydroxy-Glutaric acid | 1.12947 | 3.67625E-10 | 2.6038 | Up |
| 60 | 2-Imidazolidone-4-carboxylic acid | 1.12618 | 1.61805E-09 | 0.5034 | Down |
| 61 | Galactose | 1.12518 | 1.4999E-09 | 2.3618 | Up |
| 62 | Allose | 1.122 | 1.14271E-09 | 0.28858 | Down |
| 63 | Inositol | 1.12105 | 5.55659E-09 | 0.54585 | Down |
| 64 | 3-hydroxy-Butanoic acid | 1.1173 | 3.54608E-09 | 2.7127 | Up |
| 65 | 9,12-Octadecadienoic acid | 1.11678 | 4.87542E-09 | 0.39534 | Down |
| 66 | Xylobiose | 1.11333 | 4.87542E-09 | 0.39534 | Down |
| 67 | 9-Octadecenoic acid | 1.11148 | 1.69278E-08 | 0.21623 | Down |
| 68 | Mannitol | 1.10758 | 1.25935E-08 | 5.0245 | Up |
| 69 | Histidine | 1.10642 | 2.40661E-08 | 7.5979 | Up |
| 70 | Pantothenic acid | 1.10457 | 3.22658E-08 | 0.61203 | Down |
| 71 | Sphingosine | 1.10118 | 4.53232E-08 | 0.48163 | Down |
| 72 | Lumichrome | 1.09924 | 2.92983E-08 | 0.27694 | Down |
| 73 | myo-Inositol-2-phosphate | 1.09896 | 2.3108E-08 | 0.37153 | Down |
| 74 | Dodecanoic acid | 1.09645 | 5.08739E-08 | 0.37748 | Down |
| 75 | 4-hydroxy-Quinazoline | 1.09448 | 4.77294E-08 | 2.2612 | Up |
| 76 | Eicosatetraenoic acid | 1.09199 | 5.04174E-08 | 0.28017 | Down |
| 77 | Glycine | 1.09184 | 1.10217E-07 | 0.2025 | Down |
| 78 | Gulonic acid | 1.0913 | 6.44071E-08 | 0.39584 | Down |
| 79 | Tyrosine | 1.08973 | 1.52579E-07 | 0.44294 | Down |
| 80 | n-Tridecan-1-ol | 1.08189 | 2.32056E-07 | 0.44447 | Down |
| 81 | Xanthosine | 1.06584 | 7.27973E-07 | 1.559 | Up |
| 82 | Gentiobiose | 1.02468 | 7.48136E-06 | 10.797 | Up |
| 83 | n-Heneicosan-1-ol | 1.01183 | 1.48538E-05 | 0.55597 | Down |
| 84 | Arginine | 1.00419 | 2.02568E-05 | 0.55823 | Down |

**Table S7. Differential metabolites in response to DN2 group vs. DN1 group.**

| No | Metabolite | VIP | t-test P | FC | Trend |
| --- | --- | --- | --- | --- | --- |
| 1 | Gluconic acid | 1.52971 | 3.4E-14 | 0.15319 | Down |
| 2 | Uric acid | 1.52659 | 1.51E-13 | 2.3714 | Up |
| 3 | beta-Homoserine | 1.52551 | 2.06E-13 | 0.38941 | Down |
| 4 | Erythronic acid | 1.52262 | 4.83E-13 | 0.44287 | Down |
| 5 | 3-hydroxy-Indole | 1.51609 | 2.764E-12 | 2.46 | Up |
| 6 | Campesterol | 1.51438 | 2.446E-12 | 2.5021 | Up |
| 7 | n-Octadecan-1-ol | 1.51223 | 2.6599E-11 | 0.44722 | Down |
| 8 | beta-Sitosterol | 1.51125 | 1.0488E-11 | 2.7722 | Up |
| 9 | Malonic acid | 1.49931 | 5.42927E-10 | 0.15204 | Down |
| 10 | Trehalose | 1.49582 | 2.73244E-10 | 2.7415 | Up |
| 11 | Cellotriose | 1.49145 | 6.67167E-10 | 12.317 | Up |
| 12 | Sorbitol | 1.49026 | 3.74717E-10 | 2.1232 | Up |
| 13 | Hydantoin | 1.48933 | 1.11822E-09 | 0.67777 | Down |
| 14 | Glucoheptonic acid | 1.48367 | 2.03875E-09 | 0.19877 | Down |
| 15 | Heptadecanoic acid | 1.47751 | 6.30343E-09 | 0.51841 | Down |
| 16 | Glyceric acid | 1.47748 | 3.81491E-09 | 0.68663 | Down |
| 17 | 2-hydroxy-Glutaric acid | 1.47428 | 3.46541E-09 | 2.2249 | Up |
| 18 | Hypotaurine | 1.47119 | 6.00889E-09 | 0.53254 | Down |
| 19 | Tryptophan | 1.47056 | 8.27264E-09 | 1.6913 | Up |
| 20 | Lyxose | 1.47056 | 5.6311E-09 | 1.7172 | Up |
| 21 | 3-hydroxy-Proline | 1.46102 | 2.9387E-08 | 1.9242 | Up |
| 22 | Glucuronic acid | 1.45438 | 3.81551E-08 | 1.5844 | Up |
| 23 | Methionine | 1.452 | 4.08674E-08 | 1.5686 | Up |
| 24 | Lysine | 1.44865 | 9.53806E-08 | 0.4338 | Down |
| 25 | Arginine | 1.44391 | 1.27711E-07 | 0.41081 | Down |
| 26 | Eicosatetraenoic acid | 1.3946 | 1.89303E-06 | 0.61752 | Down |
| 27 | Glycerol | 1.34105 | 1.75794E-05 | 0.38691 | Down |
| 28 | 3-hydroxy-Butanoic acid | 1.33472 | 8.92089E-06 | 0.67252 | Down |
| 29 | Pantothenic acid | 1.28005 | 4.47207E-05 | 0.67703 | Down |
| 30 | 1-Pyrroline-3-hydroxy-5-carboxylic acid | 1.27137 | 8.67464E-05 | 0.18476 | Down |
| 31 | Galactose | 1.2711 | 7.58928E-05 | 1.531 | Up |
| 32 | Threitol | 1.22743 | 0.000198746 | 0.56679 | Down |
| 33 | Gentiobiose | 1.18387 | 0.000589606 | 2.5442 | Up |
| 34 | 4-hydroxy-Quinazoline | 1.17988 | 0.000509277 | 1.5239 | Up |
| 35 | Thymine | 1.17973 | 0.00049195 | 0.59183 | Down |
| 36 | Nigerose | 1.176 | 0.000567727 | 1.4409 | Up |
| 37 | Benzoic acid | 1.17103 | 0.000626511 | 0.61272 | Down |
| 38 | Mannitol | 1.15694 | 0.000793043 | 1.5392 | Up |
| 39 | Lumichrome | 1.13279 | 0.001114734 | 0.62141 | Down |
| 40 | Carbodiimide | 1.07983 | 0.003002965 | 0.26534 | Down |
| 41 | Pyrophosphate | 1.06387 | 0.002198954 | 1.3546 | Up |
| 42 | Isobutanoic acid | 1.04483 | 0.004457463 | 0.59215 | Down |
| 43 | Lactic acid | 1.00851 | 0.005772222 | 1.4299 | Up |

**Table S8. Differential metabolites in response to DN2+ZnCM group vs. DN2 group.**

| No | Metabolite | VIP | t-test P | FC | Trend |
| --- | --- | --- | --- | --- | --- |
| 1 | Octadecadienoic acid | 1.32386 | <0.000000000000001 | 0.14185 | Down |
| 2 | Campesterol | 1.32206 | <0.000000000000001 | 0.30453 | Down |
| 3 | 9,12-Octadecadienoic acid | 1.31953 | 3.8E-14 | 0.43184 | Down |
| 4 | beta-Sitosterol | 1.31769 | 3.7E-13 | 0.3384 | Down |
| 5 | Arginine | 1.31576 | 5E-15 | 4.2395 | Up |
| 6 | myo-Inositol-2-phosphate | 1.31466 | 1.895E-12 | 0.54386 | Down |
| 7 | 2-hydroxy-Glutaric acid | 1.3137 | 1.185E-12 | 0.18664 | Down |
| 8 | Methionine | 1.31325 | 2.99E-13 | 0.37441 | Down |
| 9 | Gluconic acid | 1.30993 | 1.508E-12 | 3.2733 | Up |
| 10 | Glycolic acid-2-phosphate | 1.30724 | 4.524E-12 | 0.45313 | Down |
| 11 | Lysine | 1.30696 | 9.12E-13 | 7.8263 | Up |
| 12 | Rhamnose | 1.3062 | 1.1042E-11 | 6.0436 | Up |
| 13 | Erythronic acid | 1.30447 | 2.309E-12 | 4.1543 | Up |
| 14 | Trehalose | 1.30326 | 1.28908E-10 | 0.39937 | Down |
| 15 | Pinitol | 1.29828 | 2.52254E-10 | 0.46692 | Down |
| 16 | Glucose | 1.29827 | 1.40634E-10 | 0.40754 | Down |
| 17 | 4-hydroxyphenylacetic acid | 1.29596 | 1.35912E-10 | 6.1413 | Up |
| 18 | Taurine | 1.29479 | 4.7782E-11 | 2.5587 | Up |
| 19 | Carbodiimide | 1.29139 | 1.05744E-10 | 2.846 | Up |
| 20 | 3-hydroxy-Butanoic acid | 1.28969 | 5.31474E-10 | 0.21629 | Down |
| 21 | Sophorose | 1.28827 | 6.4623E-10 | 0.65955 | Down |
| 22 | 3-hydroxy-Proline | 1.28751 | 3.79701E-10 | 0.55864 | Down |
| 23 | Glyceric acid | 1.28498 | 5.07899E-10 | 2.6025 | Up |
| 24 | Glycerol-3-phosphate | 1.28482 | 3.50078E-09 | 0.62808 | Down |
| 25 | Glucuronic acid | 1.28339 | 7.06472E-10 | 3.4934 | Up |
| 26 | Galacturonic acid | 1.27725 | 1.56778E-09 | 2.6641 | Up |
| 27 | Methionine sulfoxide | 1.2737 | 7.59701E-09 | 0.70536 | Down |
| 28 | Xanthosine | 1.27174 | 1.40656E-09 | 0.44345 | Down |
| 29 | Phosphoric acid | 1.27125 | 1.17007E-08 | 0.63393 | Down |
| 30 | Phenylalanine | 1.2703 | 2.29721E-09 | 1.8139 | Up |
| 31 | n-Octadecan-1-ol | 1.26964 | 3.15467E-09 | 2.4809 | Up |
| 32 | Aspartic acid | 1.26399 | 6.88778E-09 | 1.5226 | Up |
| 33 | Creatinine | 1.24085 | 9.55978E-08 | 1.7971 | Up |
| 34 | Thymine | 1.24039 | 7.68366E-08 | 3.3979 | Up |
| 35 | Fumaric acid | 1.23512 | 2.39798E-07 | 0.42237 | Down |
| 36 | alpha-Tocopherol | 1.23217 | 3.92319E-07 | 0.53622 | Down |
| 37 | Cellotriose | 1.2294 | 4.34791E-07 | 0.32635 | Down |
| 38 | Ornithine | 1.21836 | 5.15254E-07 | 1.6623 | Up |
| 39 | Gulose | 1.2157 | 9.75266E-07 | 1.8473 | Up |
| 40 | Inositol | 1.21313 | 1.54458E-06 | 0.50684 | Down |
| 41 | Xylulose | 1.20354 | 1.95887E-06 | 0.5037 | Down |
| 42 | Lyxose | 1.20237 | 2.1759E-06 | 0.60609 | Down |
| 43 | Hypotaurine | 1.1941 | 1.50473E-06 | 1.6271 | Up |
| 44 | Arabitol | 1.19223 | 4.63316E-06 | 0.61095 | Down |
| 45 | Hydantoin | 1.19 | 1.88515E-06 | 1.4635 | Up |
| 46 | 3-hydroxy-Indole | 1.18822 | 2.11164E-06 | 1.4445 | Up |
| 47 | Gulonic acid | 1.17949 | 4.3432E-06 | 1.5371 | Up |
| 48 | Asparagine | 1.17915 | 4.4934E-06 | 1.3579 | Up |
| 49 | Calystegine B2 | 1.17553 | 1.27212E-05 | 0.67345 | Down |
| 50 | Pyruvic acid | 1.1716 | 7.81592E-06 | 0.4444 | Down |
| 51 | Lyxonic acid | 1.17 | 4.7004E-06 | 2.6566 | Up |
| 52 | Allantoin | 1.16621 | 4.36511E-06 | 7.8122 | Up |
| 53 | Tyrosine | 1.16161 | 1.10111E-05 | 1.8235 | Up |
| 54 | Histidine | 1.15383 | 9.48434E-06 | 2.0349 | Up |
| 55 | 1-Pyrroline-3-hydroxy-5-carboxylic acid | 1.14318 | 1.46773E-05 | 6.4548 | Up |
| 56 | n-Eicosan-1-ol | 1.14235 | 3.49316E-05 | 2.0468 | Up |
| 57 | Cytosine | 1.13968 | 1.08076E-05 | 1.5048 | Up |
| 58 | Galactose | 1.13633 | 1.93805E-05 | 0.66358 | Down |
| 59 | Lumichrome | 1.09367 | 8.97904E-05 | 1.6736 | Up |
| 60 | 2,4-dihydroxy-Butanoic acid | 1.07782 | 0.000139245 | 0.70547 | Down |
| 61 | 2-Imidazolidone-4-carboxylic acid | 1.06945 | 0.000173486 | 1.419 | Up |
| 62 | Sulfuric acid | 1.06305 | 0.000234177 | 0.69087 | Down |
| 63 | Nigerose | 1.04492 | 0.000262288 | 0.65139 | Down |
| 64 | Urea | 1.03363 | 0.000411305 | 5.9669 | Up |
| 65 | Pantothenic acid | 1.02605 | 0.000325984 | 1.5395 | Up |
| 66 | Benzoic acid | 1.0075 | 0.000564251 | 1.5692 | Up |
| 67 | Glutamic acid | 1.0065 | 0.000964688 | 0.5869 | Down |

**Table S9. The related dataset of** **Spearman’s rank correlations.**

|  | Urinary volume | Urine Albumin | Serum BUN | Serum creatinine | Serum urea | Serum uric acid | Bacteroidales_S24-7_group | Ruminococcaceae | [Eubacterium]_copr | Prevotella_9 | Gluconic acid | Lysine | Lumichrome | 3-Hydroxybutanoic | Glucuronic acid | Serum TNF-α | Serum IL-β | Serum IL-6 | Kidney Zn | Kidneyl Cd | Kidney Zn/Cd |
| --- | --- | --- | --- | --- | --- | --- | --- | --- | --- | --- | --- | --- | --- | --- | --- | --- | --- | --- | --- | --- | --- |
| Urinary volume | 1 | 0.82434 | 0.86912 | 0.85233 | 0.86008 | 0.90665 | -0.60744 | 0.52151 | 0 | 0 | -0.93025 | -0.71382 | -0.89573 | 0 | 0 | 0.87471 | 0.89226 | 0.85023 | -0.64778 | 0.88681 | -0.90154 |
| Urine Albumin | 0.82434 | 1 | 0.83484 | 0.80529 | 0.85385 | 0.82246 | -0.51144 | 0 | 0 | 0 | -0.83189 | -0.74165 | -0.7955 | 0 | 0 | 0.82402 | 0.86304 | 0.81182 | -0.66792 | 0.72983 | -0.79381 |
| Serum BUN | 0.86912 | 0.83484 | 1 | 0.87128 | 0.85886 | 0.90965 | -0.57095 | 0 | 0 | 0 | -0.82452 | -0.79225 | -0.84882 | 0 | 0 | 0.91552 | 0.83841 | 0.81308 | -0.66635 | 0.77809 | -0.80698 |
| Serum creatinine | 0.85233 | 0.80529 | 0.87128 | 1 | 0.92481 | 0.91763 | -0.62977 | 0 | 0 | 0 | -0.80998 | -0.73015 | -0.8036 | 0 | 0 | 0.87471 | 0.79657 | 0.8112 | -0.65134 | 0.75229 | -0.78446 |
| Serum urea | 0.86008 | 0.85385 | 0.85886 | 0.92481 | 1 | 0.90895 | -0.6 | 0 | 0 | 0 | -0.81351 | -0.79456 | -0.8561 | 0 | 0 | 0.87467 | 0.83189 | 0.8409 | -0.67917 | 0.72871 | -0.78874 |
| Serum uric acid | 0.90665 | 0.82246 | 0.90965 | 0.91763 | 0.90895 | 1 | -0.62967 | 0 | 0 | 0 | -0.83128 | -0.76448 | -0.88344 | 0 | 0 | 0.87884 | 0.85708 | 0.83691 | -0.65547 | 0.81458 | -0.83212 |
| Bacteroidales_S24-7_group | -0.60744 | -0.51144 | -0.57095 | -0.62977 | -0.6 | -0.62967 | 1 | 0 | 0 | 0 | 0.60413 | 0.5985 | 0.71088 | 0 | 0 | -0.57917 | -0.56492 | -0.56079 | 0 | -0.57598 | 0.58311 |
| Ruminococcaceae | 0.52151 | 0 | 0 | 0 | 0 | 0 | 0 | 1 | 0 | 0 | 0 | 0 | 0 | 0 | 0 | 0 | 0 | 0 | 0 | 0.5379 | -0.54015 |
| [Eubacterium]_copr | 0 | 0 | 0 | 0 | 0 | 0 | 0 | 0 | 1 | 0 | 0 | 0 | 0 | 0 | 0 | 0 | 0 | 0 | 0 | 0 | 0 |
| Prevotella_9 | 0 | 0 | 0 | 0 | 0 | 0 | 0 | 0 | 0 | 1 | 0 | 0 | 0 | -0.55434 | 0 | 0 | 0 | 0 | 0 | 0 | 0 |
| Gluconic acid | -0.93025 | -0.83189 | -0.82452 | -0.80998 | -0.81351 | -0.83128 | 0.60413 | 0 | 0 | 0 | 1 | 0.65722 | 0.86773 | 0 | 0 | -0.83358 | -0.86735 | -0.87824 | 0.70244 | -0.86792 | 0.92008 |
| Lysine | -0.71382 | -0.74165 | -0.79225 | -0.73015 | -0.79456 | -0.76448 | 0.5985 | 0 | 0 | 0 | 0.65722 | 1 | 0.76285 | -0.62214 | 0.59306 | -0.79568 | -0.77917 | -0.68124 | 0.65291 | -0.54484 | 0.60281 |
| Lumichrome | -0.89573 | -0.7955 | -0.84882 | -0.8036 | -0.8561 | -0.88344 | 0.71088 | 0 | 0 | 0 | 0.86773 | 0.76285 | 1 | 0 | 0 | -0.83809 | -0.88161 | -0.86904 | 0.69231 | -0.78218 | 0.8272 |
| 3-Hydroxybutanoic | 0 | 0 | 0 | 0 | 0 | 0 | 0 | 0 | 0 | -0.55434 | 0 | -0.62214 | 0 | 1 | -0.7 | 0 | 0 | 0 | 0 | 0 | 0 |
| Glucuronic acid | 0 | 0 | 0 | 0 | 0 | 0 | 0 | 0 | 0 | 0 | 0 | 0.59306 | 0 | -0.7 | 1 | 0 | 0 | 0 | 0.60694 | 0 | 0 |
| Serum TNF-α | 0.87471 | 0.82402 | 0.91552 | 0.87471 | 0.87467 | 0.87884 | -0.57917 | 0 | 0 | 0 | -0.83358 | -0.79568 | -0.83809 | 0 | 0 | 1 | 0.85647 | 0.81295 | -0.67674 | 0.74053 | -0.79981 |
| Serum IL-β | 0.89226 | 0.86304 | 0.83841 | 0.79657 | 0.83189 | 0.85708 | -0.56492 | 0 | 0 | 0 | -0.86735 | -0.77917 | -0.88161 | 0 | 0 | 0.85647 | 1 | 0.84615 | -0.69812 | 0.77674 | -0.82251 |
| Serum IL-6 | 0.85023 | 0.81182 | 0.81308 | 0.8112 | 0.8409 | 0.83691 | -0.56079 | 0 | 0 | 0 | -0.87824 | -0.68124 | -0.86904 | 0 | 0 | 0.81295 | 0.84615 | 1 | -0.69719 | 0.79794 | -0.8546 |
| Kidney Zn | -0.64778 | -0.66792 | -0.66635 | -0.65134 | -0.67917 | -0.65547 | 0 | 0 | 0 | 0 | 0.70244 | 0.65291 | 0.69231 | 0 | 0.60694 | -0.67674 | -0.69812 | -0.69719 | 1 | -0.55704 | 0.71032 |
| Kidneyl Cd | 0.88681 | 0.72983 | 0.77809 | 0.75229 | 0.72871 | 0.81458 | -0.57598 | 0.5379 | 0 | 0 | -0.86792 | -0.54484 | -0.78218 | 0 | 0 | 0.74053 | 0.77674 | 0.79794 | -0.55704 | 1 | -0.96248 |
| Kidney Zn/Cd | -0.90154 | -0.79381 | -0.80698 | -0.78446 | -0.78874 | -0.83212 | 0.58311 | -0.54015 | 0 | 0 | 0.92008 | 0.60281 | 0.8272 | 0 | 0 | -0.79981 | -0.82251 | -0.8546 | 0.71032 | -0.96248 | 1 |

**Table S10. The related dataset of P value’s correlation.**

|  | Urinary volume | Urine Albumin | Serum BUN | Serum creatinine | Serum urea | Serum uric acid | Bacteroidales_S24-7_group | Ruminococcaceae | [Eubacterium]_copr | Prevotella_9 | Gluconic acid | Lysine | Lumichrome | 3-Hydroxybutanoic | Glucuronic acid | Serum TNF-a | Serum IL-B | Serum IL-6 | Kidney Zn | Kidneyl Cd | Kidney Zn/Cd |
| --- | --- | --- | --- | --- | --- | --- | --- | --- | --- | --- | --- | --- | --- | --- | --- | --- | --- | --- | --- | --- | --- |
| Urinary volume | NA | 6.28E-11 | 3.53E-13 | 3.00E-12 | 1.16E-12 | 8.88E-16 | 3.23E-05 | 0.000559 | 0.42525 | 0.40786 | 0 | 2.33E-07 | 6.00E-15 | 0.73271 | 0.31 | 1.62E-13 | 1.07E-14 | 3.86E-12 | 6.23E-06 | 2.62E-14 | 2.22E-15 |
| Urine Albumin | 6.28E-11 | NA | 2.15E-11 | 3.72E-10 | 2.50E-12 | 7.55E-11 | 0.000744 | 0.015272 | 0.17925 | 0.98258 | 2.92E-11 | 4.37E-08 | 8.61E-10 | 0.21029 | 0.092495 | 6.48E-11 | 7.93E-13 | 2.07E-10 | 2.49E-06 | 9.12E-08 | 9.91E-10 |
| Serum BUN | 3.53E-13 | 2.15E-11 | NA | 2.63E-13 | 1.35E-12 | 4.44E-16 | 0.000119 | 0.002093 | 0.14339 | 0.90623 | 6.17E-11 | 1.13E-09 | 4.55E-12 | 0.078425 | 0.049843 | 0 | 1.46E-11 | 1.84E-10 | 2.68E-06 | 3.45E-09 | 3.20E-10 |
| Serum creatinine | 3.00E-12 | 3.72E-10 | 2.63E-13 | NA | 0 | 0 | 1.34E-05 | 0.002737 | 0.60487 | 0.87693 | 2.44E-10 | 8.95E-08 | 4.31E-10 | 0.15331 | 0.079362 | 1.62E-13 | 7.88E-10 | 2.19E-10 | 5.32E-06 | 2.18E-08 | 2.11E-09 |
| Serum urea | 1.16E-12 | 2.50E-12 | 1.35E-12 | 0 | NA | 4.44E-16 | 4.27E-05 | 0.009161 | 0.22533 | 0.88033 | 1.77E-10 | 9.31E-10 | 1.90E-12 | 0.090856 | 0.039192 | 1.63E-13 | 2.92E-11 | 1.12E-11 | 1.45E-06 | 9.76E-08 | 1.50E-09 |
| Serum uric acid | 8.88E-16 | 7.55E-11 | 4.44E-16 | 0 | 4.44E-16 | NA | 1.34E-05 | 0.001978 | 0.35221 | 0.78836 | 3.11E-11 | 9.39E-09 | 4.44E-14 | 0.12439 | 0.070658 | 8.90E-14 | 1.69E-12 | 1.72E-11 | 4.43E-06 | 1.60E-10 | 2.85E-11 |
| Bacteroidales_S24-7_group | 3.23E-05 | 0.000744 | 0.000119 | 1.34E-05 | 4.27E-05 | 1.34E-05 | NA | 0.15196 | 0.11733 | 0.82444 | 3.66E-05 | 4.52E-05 | 2.75E-07 | 0.12604 | 0.11705 | 9.02E-05 | 0.000146 | 0.000167 | 0.001067 | 0.000101 | 7.86E-05 |
| Ruminococcaceae | 0.000559 | 0.015272 | 0.002093 | 0.002737 | 0.009161 | 0.001978 | 0.15196 | NA | 0.049854 | 0.42762 | 0.001264 | 0.047026 | 0.008348 | 0.48339 | 0.32219 | 0.003429 | 0.001133 | 0.013911 | 0.001701 | 0.000344 | 0.000321 |
| [Eubacterium]_copr | 0.42525 | 0.17925 | 0.14339 | 0.60487 | 0.22533 | 0.35221 | 0.11733 | 0.049854 | NA | 0.12587 | 0.24633 | 0.10306 | 0.19886 | 0.25411 | 0.071922 | 0.39938 | 0.14292 | 0.30752 | 0.067758 | 0.4307 | 0.26155 |
| Prevotella_9 | 0.40786 | 0.98258 | 0.90623 | 0.87693 | 0.88033 | 0.78836 | 0.82444 | 0.42762 | 0.12587 | NA | 0.55154 | 0.049267 | 0.80435 | 0.000206 | 0.006566 | 0.75448 | 0.89714 | 0.82444 | 0.32217 | 0.19801 | 0.37346 |
| Gluconic acid | 0 | 2.92E-11 | 6.17E-11 | 2.44E-10 | 1.77E-10 | 3.11E-11 | 3.66E-05 | 0.001264 | 0.24633 | 0.55154 | NA | 4.09E-06 | 4.27E-13 | 0.69661 | 0.4273 | 2.45E-11 | 4.49E-13 | 9.73E-14 | 4.37E-07 | 4.16E-13 | 0 |
| Lysine | 2.33E-07 | 4.37E-08 | 1.13E-09 | 8.95E-08 | 9.31E-10 | 9.39E-09 | 4.52E-05 | 0.047026 | 0.10306 | 0.049267 | 4.09E-06 | NA | 1.05E-08 | 1.82E-05 | 5.51E-05 | 8.48E-10 | 3.18E-09 | 1.31E-06 | 4.96E-06 | 0.000278 | 3.85E-05 |
| Lumichrome | 6.00E-15 | 8.61E-10 | 4.55E-12 | 4.31E-10 | 1.90E-12 | 4.44E-14 | 2.75E-07 | 0.008348 | 0.19886 | 0.80435 | 4.27E-13 | 1.05E-08 | NA | 0.19845 | 0.039192 | 1.52E-11 | 5.88E-14 | 3.57E-13 | 7.47E-07 | 2.52E-09 | 4.72E-11 |
| 3-Hydroxybutanoic | 0.73271 | 0.21029 | 0.078425 | 0.15331 | 0.090856 | 0.12439 | 0.12604 | 0.48339 | 0.25411 | 0.000206 | 0.69661 | 1.82E-05 | 0.19845 | NA | 4.98E-07 | 0.098533 | 0.29273 | 0.38967 | 0.10003 | 0.5143 | 0.72407 |
| Glucuronic acid | 0.31 | 0.092495 | 0.049843 | 0.079362 | 0.039192 | 0.070658 | 0.11705 | 0.32219 | 0.071922 | 0.006566 | 0.4273 | 5.51E-05 | 0.039192 | 4.98E-07 | NA | 0.03266 | 0.078959 | 0.33027 | 3.29E-05 | 0.9725 | 0.45278 |
| Serum TNF-a | 1.62E-13 | 6.48E-11 | 0 | 1.62E-13 | 1.63E-13 | 8.90E-14 | 9.02E-05 | 0.003429 | 0.39938 | 0.75448 | 2.45E-11 | 8.48E-10 | 1.52E-11 | 0.098533 | 0.03266 | NA | 1.82E-12 | 1.86E-10 | 1.63E-06 | 4.70E-08 | 5.98E-10 |
| Serum IL-B | 1.07E-14 | 7.93E-13 | 1.46E-11 | 7.88E-10 | 2.92E-11 | 1.69E-12 | 0.000146 | 0.001133 | 0.14292 | 0.89714 | 4.49E-13 | 3.18E-09 | 5.88E-14 | 0.29273 | 0.078959 | 1.82E-12 | NA | 6.18E-12 | 5.50E-07 | 3.82E-09 | 7.51E-11 |
| Serum IL-6 | 3.86E-12 | 2.07E-10 | 1.84E-10 | 2.19E-10 | 1.12E-11 | 1.72E-11 | 0.000167 | 0.013911 | 0.30752 | 0.82444 | 9.73E-14 | 1.31E-06 | 3.57E-13 | 0.38967 | 0.33027 | 1.86E-10 | 6.18E-12 | NA | 5.78E-07 | 7.02E-10 | 2.29E-12 |
| Kidney Zn | 6.23E-06 | 2.49E-06 | 2.68E-06 | 5.32E-06 | 1.45E-06 | 4.43E-06 | 0.001067 | 0.001701 | 0.067758 | 0.32217 | 4.37E-07 | 4.96E-06 | 7.47E-07 | 0.10003 | 3.29E-05 | 1.63E-06 | 5.50E-07 | 5.78E-07 | NA | 0.000189 | 2.84E-07 |
| Kidneyl Cd | 2.62E-14 | 9.12E-08 | 3.45E-09 | 2.18E-08 | 9.76E-08 | 1.60E-10 | 0.000101 | 0.000344 | 0.4307 | 0.19801 | 4.16E-13 | 0.000278 | 2.52E-09 | 0.5143 | 0.9725 | 4.70E-08 | 3.82E-09 | 7.02E-10 | 0.000189 | NA | 0 |
| Kidney Zn/Cd | 2.22E-15 | 9.91E-10 | 3.20E-10 | 2.11E-09 | 1.50E-09 | 2.85E-11 | 7.86E-05 | 0.000321 | 0.26155 | 0.37346 | 0 | 3.85E-05 | 4.72E-11 | 0.72407 | 0.45278 | 5.98E-10 | 7.51E-11 | 2.29E-12 | 2.84E-07 | 0 | NA |
