# Supplementary material for: Zn(II)–curcumin prevents cadmium-aggravated diabetic nephropathy by regulating gut microbiota and zinc homeostasis
Source: Front Pharmacol. 2024 Jun 5;15:1411230. doi: 10.3389/fphar.2024.1411230 (PMC11188322; doi:10.3389/fphar.2024.1411230)
Supplement: Supplementary file 2 [file DataSheet1.doc]

**Supplementary Figures**

**Control**

**
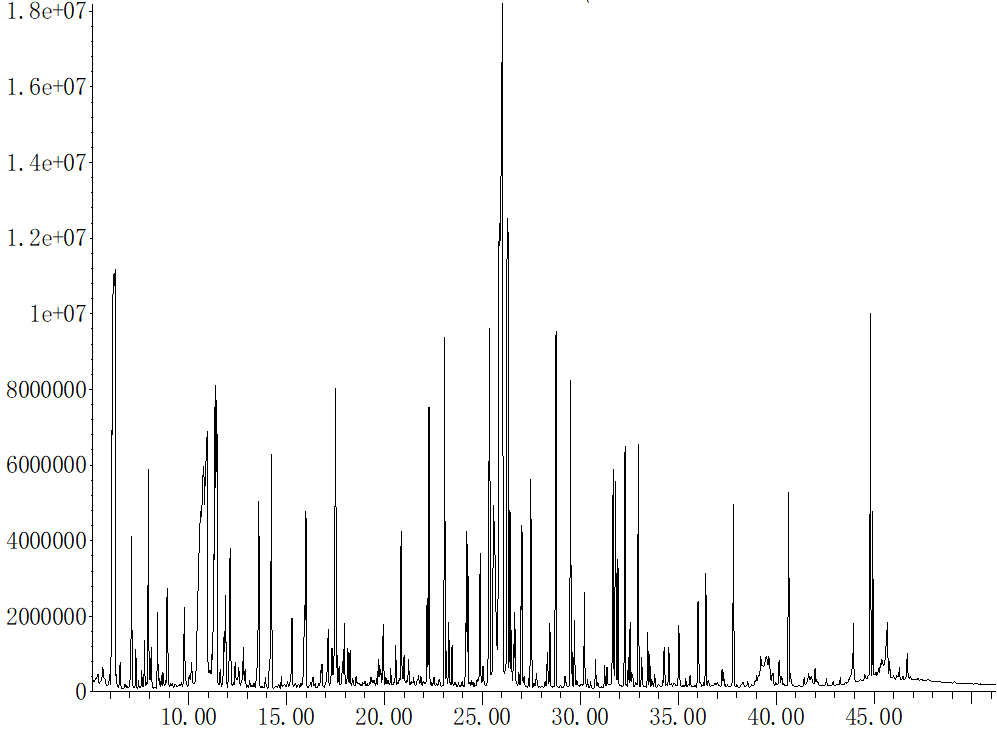
**

**DN1**

**
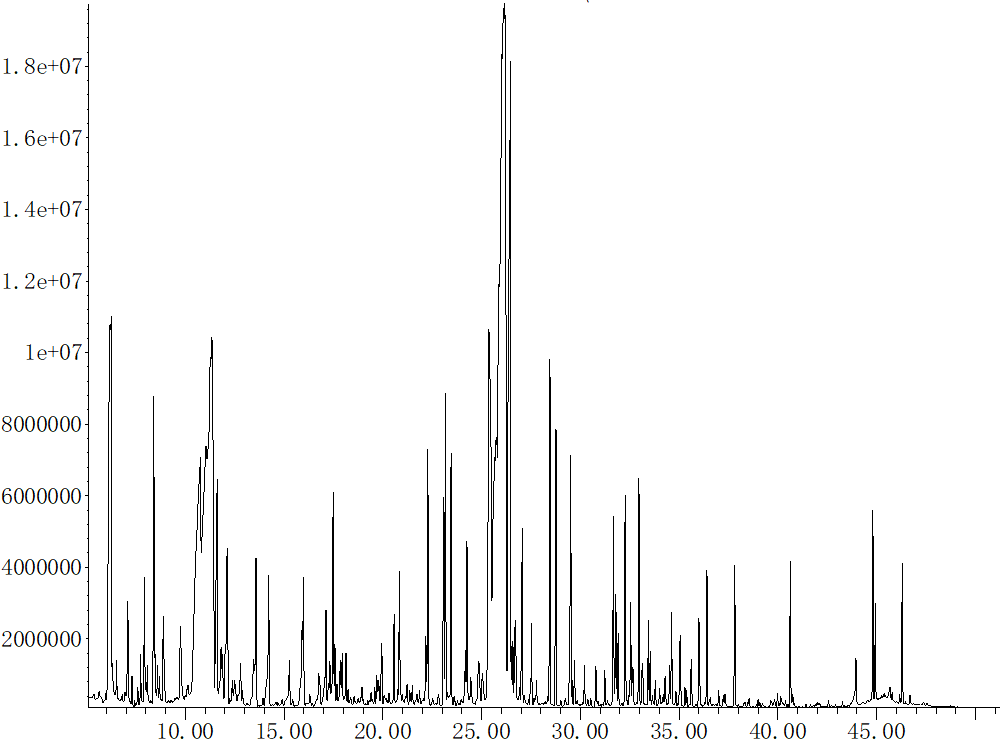
**

**DN1+ZnCM**

**
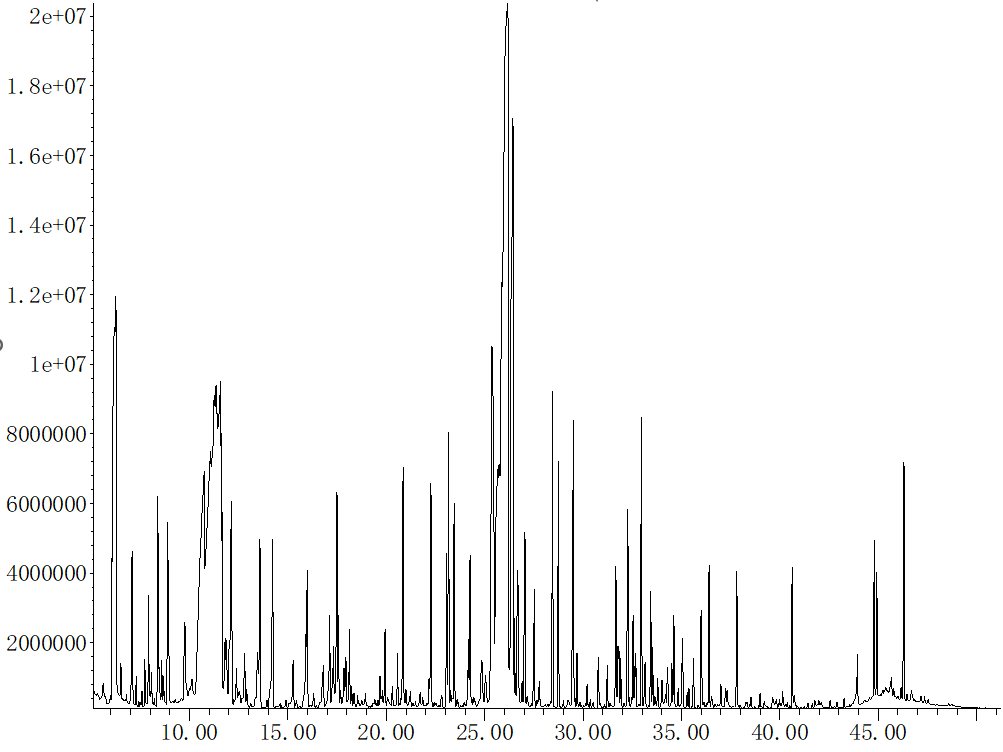
**

**DN2**

**
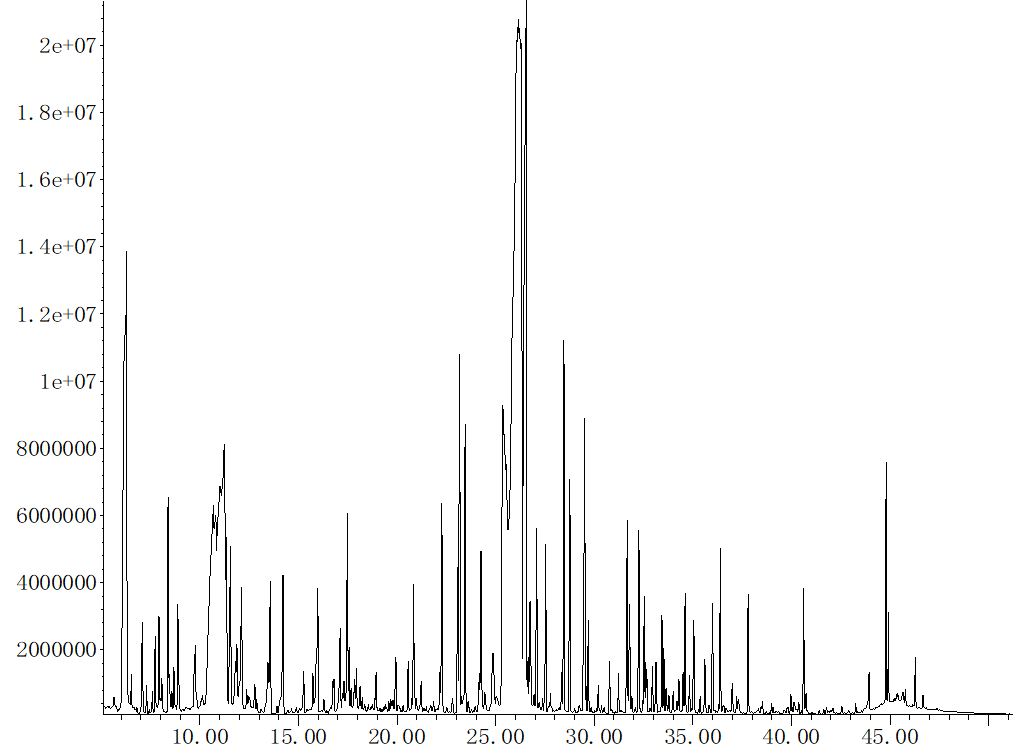
**

**DN2+ZnCM**

**
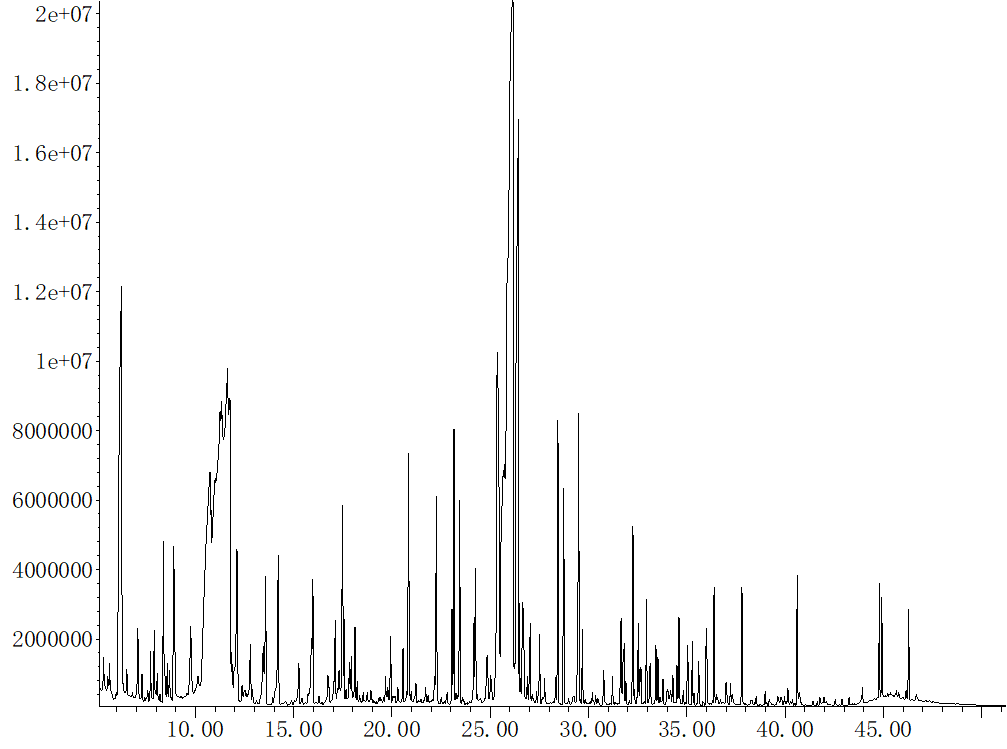
**

**FIGURE S1. Representative total ion chromatograms (TICs) of serum samples.**


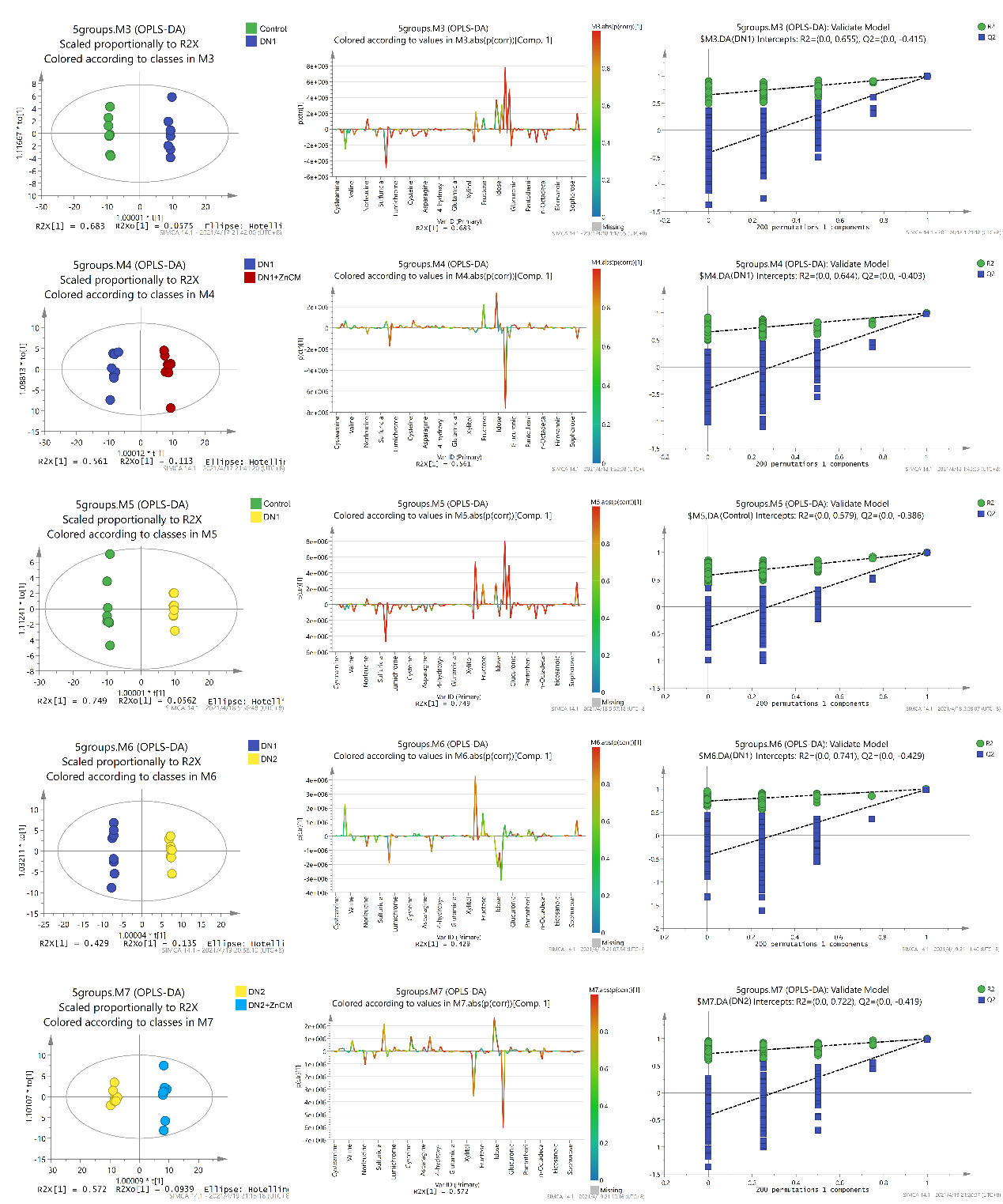


**FIGURE S2. OPLS-DA score plots and S-line plots for discriminating the metabolite and corresponding permutation test (200 times) obtained from GC-MS.**


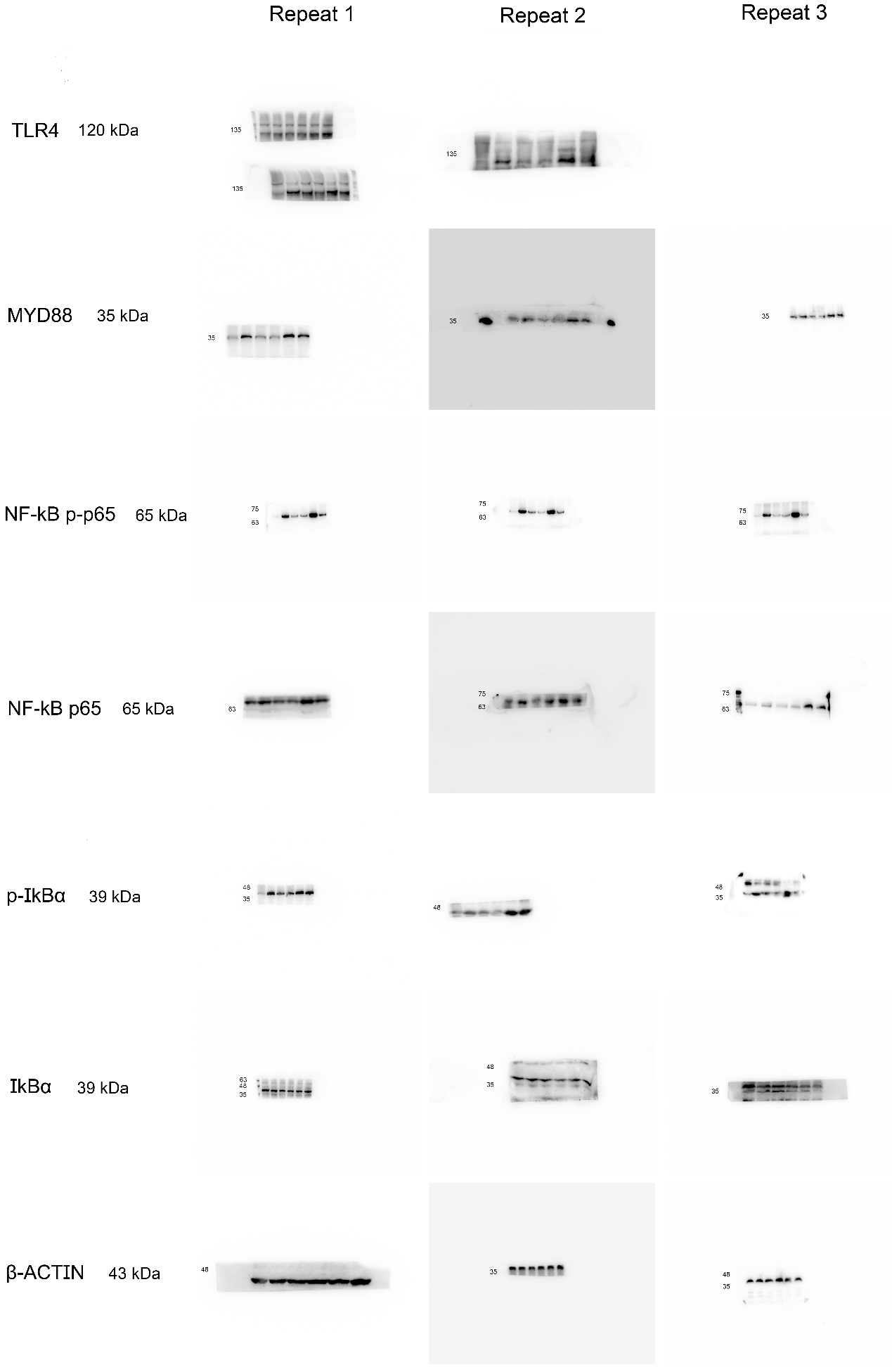

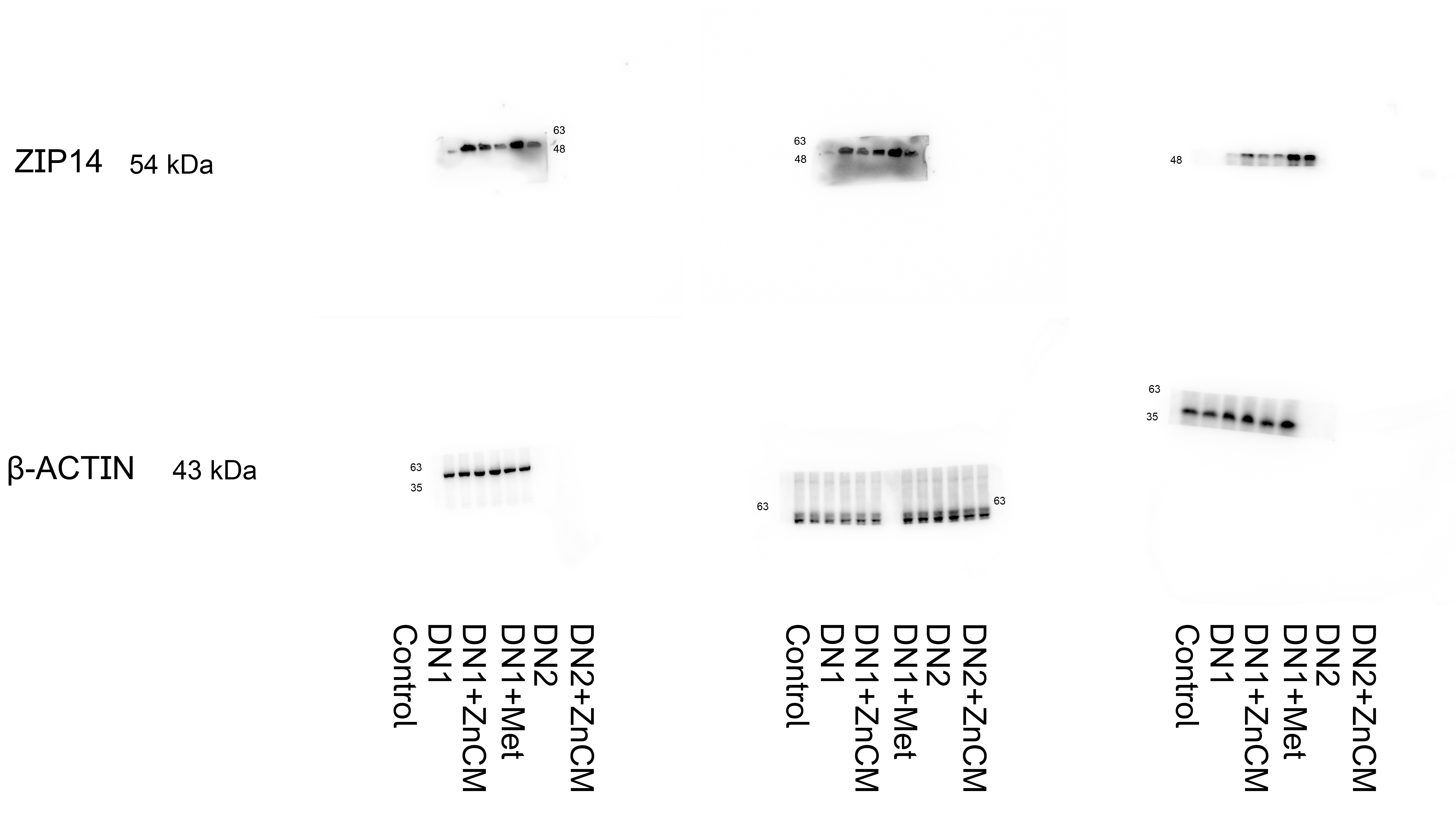


**FIGURE S3. The whole un-cropped images of the original western blots**
